# Synaptic loss and its association with symptom severity in Parkinson’s disease

**DOI:** 10.1038/s41531-024-00655-9

**Published:** 2024-02-24

**Authors:** Sophie E. Holmes, Praveen Honhar, Sule Tinaz, Mika Naganawa, Ansel T. Hilmer, Jean-Dominique Gallezot, Mark Dias, Yanghong Yang, Takuya Toyonaga, Irina Esterlis, Adam Mecca, Christopher Van Dyck, Shannan Henry, Jim Ropchan, Nabeel Nabulsi, Elan D. Louis, Robert Comley, Sjoerd J. Finnema, Richard E. Carson, David Matuskey

**Affiliations:** 1 Department of Psychiatry, Yale School of Medicine, New Haven, CT USA; 2 Department of Neurology, Yale School of Medicine, New Haven, CT USA; 3 Radiology and Biomedical Imaging, Yale School of Medicine, New Haven, CT USA; 4 Department of Biomedical Engineering, Yale School of Engineering and Applied Sciences, Yale University, New Haven, CT USA; 5 Department of Neurology, University of Texas Southwestern Medical Center, New Haven, CT USA; 6 AbbVie, North Chicago, IL USA

**Keywords:** Parkinson's disease, Motor control

## Abstract

Parkinson’s disease (PD) is the fastest growing neurodegenerative disease, but at present there is no cure, nor any disease-modifying treatments. Synaptic biomarkers from in vivo imaging have shown promise in imaging loss of synapses in PD and other neurodegenerative disorders. Here, we provide new clinical insights from a cross-sectional, high-resolution positron emission tomography (PET) study of 30 PD individuals and 30 age- and sex-matched healthy controls (HC) with the radiotracer [^11^C]UCB-J, which binds to synaptic vesicle glycoprotein 2A (SV2A), and is therefore, a biomarker of synaptic density in the living brain. We also examined a measure of relative brain perfusion from the early part of the same PET scan. Our results provide evidence for synaptic density loss in the substantia nigra that had been previously reported, but also extend this to other early-Braak stage regions known to be affected in PD (brainstem, caudate, olfactory cortex). Importantly, we also found a direct association between synaptic density loss in the nigra and severity of symptoms in patients. A greater extent and wider distribution of synaptic density loss in PD patients with longer illness duration suggests that [^11^C]UCB-J PET can be used to measure synapse loss with disease progression. We also demonstrate lower brain perfusion in PD vs. HC groups, with a greater extent of abnormalities in those with longer duration of illness, suggesting that [^11^C]UCB-J PET can simultaneously provide information on changes in brain perfusion. These results implicate synaptic imaging as a useful PD biomarker for future disease-modifying interventions.

## Introduction

Parkinson’s disease (PD) is the fastest growing neurological disorder and due to an aging population, the number of people affected is poised for exponential growth. At present, there are no approved pharmacological interventions that modify the disease process or slow its progression. Discovery of a cure will depend on a more complete understanding of the underlying disease-mechanisms and on the ability to track how novel disease-modifying interventions affect these mechanisms. PD is characterized by a toxic accumulation of misfolded α-synuclein aggregates (Lewy bodies) in presynaptic terminals, which are thought to propagate from cell-to-cell and ultimately cause neurodegeneration^1^. The prominent (Braak staging) model of PD progression posits that α-synuclein spreads in a specific pattern - first affecting regions of the brainstem and substantia nigra, then spreading to the striatum and cortical areas - a pattern that reflects how both motor and non-motor symptoms manifest as the disease progresses^2^. Two progression subtypes have been proposed based on the origin of α-synuclein – a ‘brain-first’ subtype with α-synuclein originating in the brain or the olfactory bulb, and a ‘body-first’ subtype, where α-synuclein enters the brain via the vagus nerve^3^. Detecting and tracking the synaptic loss that occurs alongside α-synuclein will be crucial in further advancing the mechanistic understanding of PD across different stages - from prodromal to advanced - and in evaluating the ability of new treatments to slow, and ultimately halt, disease progression. We can image synapses in the living brain with positron emission tomography (PET) and the radioligand [^11^C]UCB-J, which binds to the synaptic vesicle glycoprotein 2A (SV2A) – a protein ubiquitously expressed on all synaptic vesicles that is used as a marker of synaptic density^4^.

We previously used [^11^C]UCB-J PET to provide the first in vivo evidence of synaptic density loss in people with PD, observing almost 50% lower synaptic density in the substantia nigra in PD^5^. Subsequent studies largely replicated these findings, confirming lower synaptic density in substantia nigra^6,7^.

Important unanswered questions are the clinical relevance of synaptic imaging in PD, i.e., its relationship with disease severity, and whether synaptic imaging can track disease progression, which will be crucial in its utility as a biomarker and in the evaluation of disease-modifying interventions. A recent longitudinal study showed no difference in synaptic density between baseline and +2 year follow up, despite a worsening of motor symptoms, suggesting that synaptic imaging may not be sensitive enough to track disease progression in such a narrow window or in early stages of the disease^8^. Another study showed synaptic loss in cortical association areas in PD dementia and Lewy-body dementia, which also correlated with worse cognitive functioning^9^. These findings add to evidence that synaptic density is first reduced in the substantia nigra, and that pathology then spreads to cortical regions as the disease progresses and patients develop severe cognitive impairment, as posed by the Braak staging hypothesis based on α-synuclein staining^10^.

Central to the value of an imaging marker of PD progression is its association with symptom severity. To date, only one [^11^C]UCB-J PET study has demonstrated an association between synaptic density (in lower brainstem) and symptom (motor) severity, specifically in early PD^7^. The general lack of association with clinical measures may be due to relatively small samples, noise in clinical measures and a narrow window of disease duration (generally early PD, within 2 years of diagnosis). Examining whether synaptic loss is more prominent and/or widespread in patients with a longer duration of illness will shed new light on a) the progression of synaptic density loss in PD as it relates to clinical presentation and b) the ability of synaptic imaging to track disease progression.

In addition to measuring synaptic loss, [^11^C]UCB-J PET can be utilized to derive ‘*R*_1_’ - a proxy for relative brain perfusion (delivery of blood to the brain – a surrogate marker of neuronal activity made possible by the high extraction of [^11^C]UCB-J from blood). [^11^C]UCB-J PET has been used to demonstrate changes in brain perfusion in the primary visual cortex in response to visual stimulation^11^, and to show patterns of brain perfusion that align with the regional metabolic patterns seen with [^18^F]FDG in Alzheimer’s disease^12^. In PD, [^18^F]FDG PET has been used to identify a distinct metabolic topography characterized by relative hypometabolism in parieto-occipital and prefrontal regions, and relative hypermetabolism in the cerebellum, pons and thalamus^13–15^. The expression of these metabolic networks in PD correlates with motor symptoms and is associated with disease progression and loss of presynaptic dopamine terminals, such that it has been proposed as a network imaging biomarker in PD. We investigate whether *R*_1_ from [^11^C]UCB-J PET can be used as a surrogate to probe blood flow changes in PD, *in addition to* synaptic loss, for the first time.

In this study, we quantify the pattern of synaptic loss in PD in a large sample, across a wide range of disease duration, allowing us to assess the association between synaptic loss and disease severity, and to evaluate the use of synaptic imaging as a clinically relevant biomarker in PD.

## Results

### Study participants: demographic information

Thirty individuals with PD (mean ± SD age = 63 ± 8; 17 women, 13 men) and 30 healthy controls (mean ± SD age = 61 ± 9; 17 women, 13 men) were imaged with [^11^C]UCB-J PET (see Table 1 for sample characteristics). PD diagnosis was confirmed using the Movement Disorders Society (MDS) diagnostic criteria^16^, and symptoms were assessed according to the MDS - Unified Parkinson’s Disease Rating Scale (MDS-UPDRS)^17^, and disease staging was determined using the Hoehn and Yahr Scale^18^. PD medications and total L-dopa equivalent daily dose are listed in Supplementary Table 1. PD patients withheld all dopaminergic PD medications on the morning of the scan such that their last dose was at least 12 h prior to [^11^C]UCB-J PET. All PD neurological exams were performed by a movement disorders neurologist in the medication ‘OFF’ state, consistent with PET scans.

**Table 1:** Demographics, clinical characteristics, and radiotracer details

	Healthy controls	Parkinson’s disease	*p*-value
N	30	30	–
Age	61.0 ± 8.8	62.8 ± 7.7	0.40
Sex (F:M)	17:13	17:13	–
MDS-UPDRS III	–	29.7 ± 8.9	–
MDS-UPDRS Total	–	48.4 ± 16.8	–
MoCA	–	26.5 ± 2.5	
Hoehn & Yahr	–	2.0 ± 0.0	–
Disease duration (since symptom onset)	–	4.6 ± 3.0	–
Injected dose (MBq)	577 ± 163	580 ± 158	0.95
Injected mass (ng/kg)	23 ± 17	21 ± 19	0.60

Reported values are Mean ± SD.

All participants underwent a comprehensive medical history, physical examination, neurological examination, routine blood tests and electrocardiogram (ECG). Exclusion criteria were any current or past clinically significant medical or neurological illness (other than PD) that could affect study outcome, cognitive impairment (as determined by a cut-off of <21 on the Montreal Cognitive Assessment (MoCA) test^19^), a history of alcohol or substance abuse, medication affecting SV2A binding (e.g., levetiracetam), current pregnancy (as documented by pregnancy testing at screening and on the day of PET imaging), breast feeding, and contraindications to magnetic resonance imaging (MRI). The study was approved by the Yale University Human Investigation Committee and Radioactive Drug Research Committee. All participants provided written informed consent before inclusion. All imaging procedures were performed at the Yale PET and MRI research facilities.

In a secondary (exploratory) analysis, we compared PD patients with longer duration of illness – specifically, those with >6 years since onset of motor symptoms – to HCs. Generally, early stage PD is considered to be within 5 years of symptom onset^20,21^, therefore we chose 6 years since symptom onset as a cut-off for a longer illness subgroup, allowing assessment of SV2A density beyond the ‘early stage’ window. The main purpose of this analysis was to examine whether the extent and spatial distribution of synaptic loss was larger in this longer illness subgroup. Clinical and demographic characteristics of this sub-group is shown in Table 2.

**Table 2:** Characteristics of the ‘longer illness’ (>6 year) PD group

	Entire PD Cohort	>6 years PD	*p*-value
*N*	30	9	–
Age	62.8 ± 7.7	63.6 ± 8.3	0.79
Sex (F:M)	17:13	4:5	–
MDS-UPDRS III	29.7 ± 8.9	35.2 ± 9.6	0.12
MDS-UPDRS Total	48.4 ± 16.8	59.2 ± 15.3	0.09
MoCA	26.5 ± 2.5	26.8 ± 2.7	0.80
Hoehn & Yahr	2.0 ± 0.0	2.0 ± 0.0	–
Disease duration (since symptom onset)	4.6 ± 3.0	8.4 ± 2.1	0.001
Injected dose (MBq)	580 ± 158	605 ± 165	0.68
Injected mass (ng/kg)	23 ± 17	21 ± 13	0.74

Reported values are Mean ± SD.

### Lower synaptic (SV2A) density in PD vs. HC

**Fig. 1:**
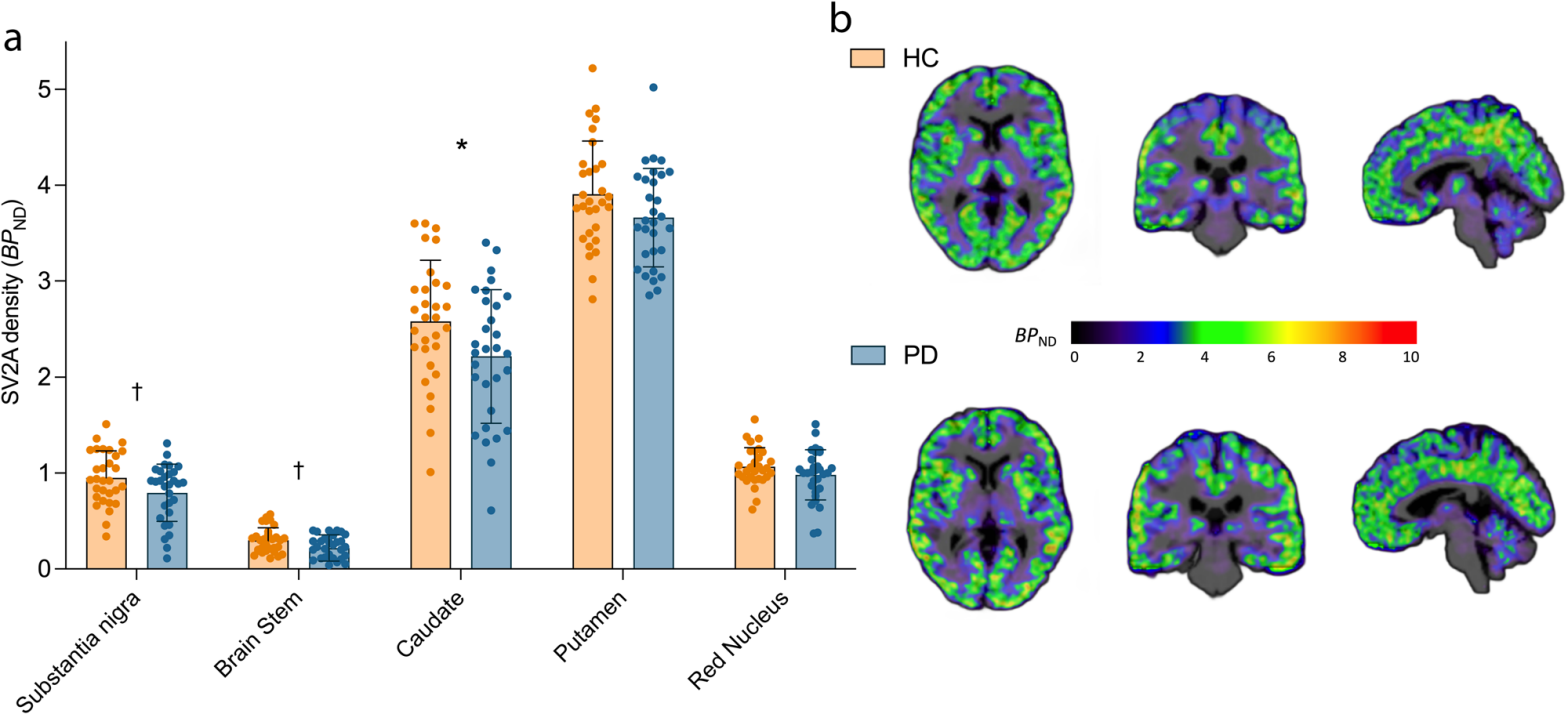
Lower synaptic density (SV2A *BP*_ND_) in PD vs. HC. **a** Synaptic density (SV2A *BP*_ND_) across *apriori* selected primary regions in HC (*n* = 30, orange) vs. PD (*n* = 30, blue). Error bars represent standard deviation. *significant after Bonferroni correction (*p* < 0.01), † significant at *p* < 0.05 (uncorrected) based on post-hoc *t-*tests (two-tailed). **b** Representative parametric *BP*_ND_ images overlaid on anatomical (T1) images in MNI space for a HC (top) and an individual with PD (bottom).

PET measurement of SV2A density expressed in the units of binding potential (*BP*_ND_) was used as a surrogate marker of synaptic density. Early Braak stage regions (substantia nigra, brainstem, red nucleus, caudate and putamen) were selected as the *apriori* primary regions for investigation, while many other cortical and subcortical regions were studied in post-hoc analyses (see, *Materials and Methods*). A linear mixed model (see, *Materials and Methods: Statistics*) showed significant effect of diagnosis on synaptic density, with the PD group (*n* = 30) exhibiting lower synaptic density (SV2A *BP*_ND_) compared to HCs (*n* = 30) across the primary brain regions (F_1,58_ = 8.72, *p* = 0.004). Post-hoc *t-*tests showed significantly lowered synaptic density in PD in the substantia nigra (−17%, *p* = 0.039), brainstem (−27%, *p* = 0.02) and caudate (−11%, *p* = 0.006), but not putamen (−3%, *p* = 0.34) or red nucleus (−8%, *p* = 0.15) (Fig. 1). Only the synaptic loss in caudate survived Bonferroni correction threshold for multiple comparisons (*p* < 0.01), but effect sizes for group differences in all *apriori* selected regions increased when we compared HC to a PD sub-cohort that only included subjects with longer illness duration (see, Results: *Findings from analysis of longer illness (>6* *yr) PD subgroup*, Supplementary Tables 2 and 3), suggesting that the measured synaptic losses in the nigra and brainstem are unlikely to be false positives. Of the exploratory regions, synaptic density in the olfactory cortex (−10%, *p* = 0.017), parahippocampal gyrus (−9%, *p* = 0.046), orbitofrontal (=8%, *p* = 0.029) and ventromedial prefrontal (−7%, *p* = 0.049) cortices was significantly lower in PD vs. HC. None of these differences in the exploratory ROIs survived Bonferroni correction. Regional *BP*_ND_ values are shown for all primary and exploratory regions in Supplementary Table 2. No hemispherical asymmetries were detected in regional synaptic densities in PD subjects.

### Lower synaptic (SV2A) density associated with worse motor function in the PD cohort

**Fig. 2:**
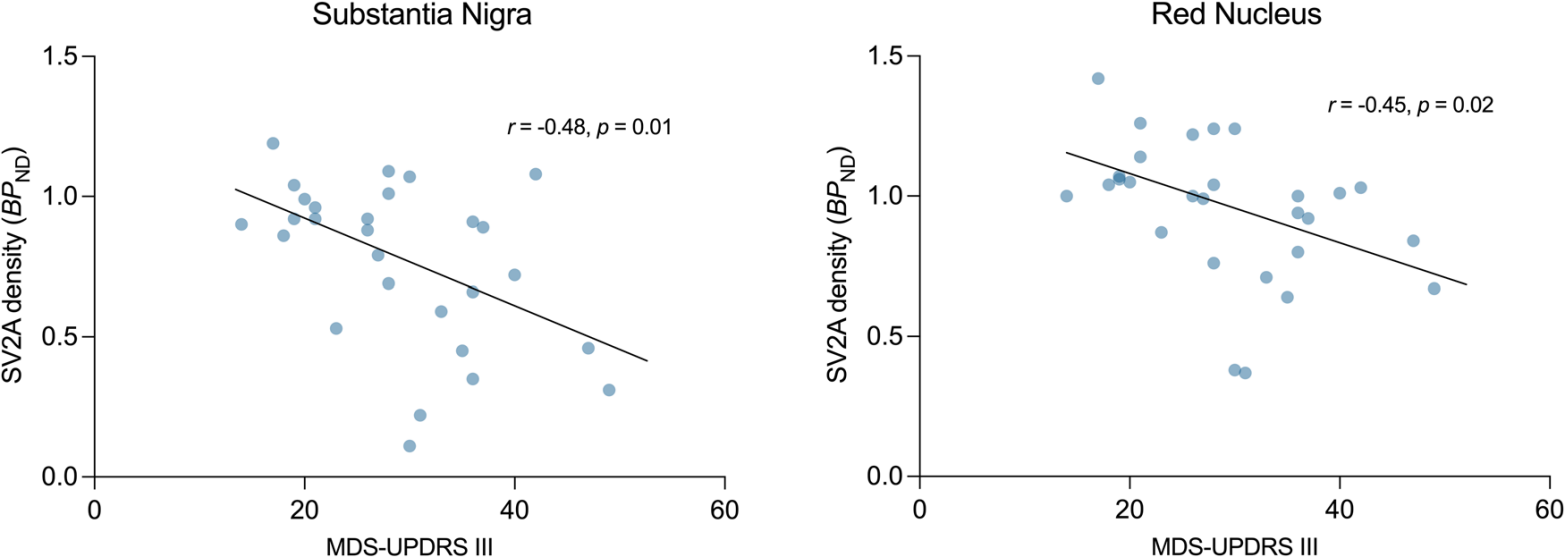
Correlation between synaptic density (SV2A *BP*_ND_) and motor function in the PD cohort. Correlations were computed using Pearson’s *r*. Each blue dot represents an individual with PD (*n* = 28). Only the correlation between substantia nigra *BP*_ND_ and MDS-UPDRS Part III scores met the Bonferroni threshold (*p* = 0.01).

Part III (motor examination) of the MDS-UPDRS (Movement Disorders Society – Unified Parkinson’s Disease Rating Scale) was significantly negatively correlated with synaptic density (SV2A *BP*_ND_) in the substantia nigra (*r* = −0.48, *p* = 0.01) and red nucleus (*r* = −0.44, *p* = 0.02), such that the lower synaptic density in these regions was associated with worse motor function (Fig. 2). Only the correlation in the substantia nigra met the threshold for Bonferroni correction (*p* = 0.01). There were also significant negative correlations between total MDS- UPDRS scores and synaptic density in both the substantia nigra (*r* = −0.45, *p* = 0.02) and red nucleus (*r* = −0.39, *p* = 0.04, Supplementary Fig. 1). There were no correlations between the clinical measures and synaptic density in any other region.

### Associations between disease duration, disease severity and synaptic (SV2A) density

PD disease duration (defined from symptom onset) was significantly associated with severity of motor function impairment (MDS-UPDRS-III, *r* = 0.43, *p* = 0.02) and total disease severity (MDS-UPDRS-Total, *r* = 0.51, *p* = 0.003) (Supplementary Fig. 2). Synaptic density in the substantia nigra showed a non-significant negative correlation with disease duration (*r* = −0.24, *p* = 0.21, Supplementary Fig. 3).

### Findings from analysis of longer illness (>6 yr) PD subgroup

**Fig. 3:**
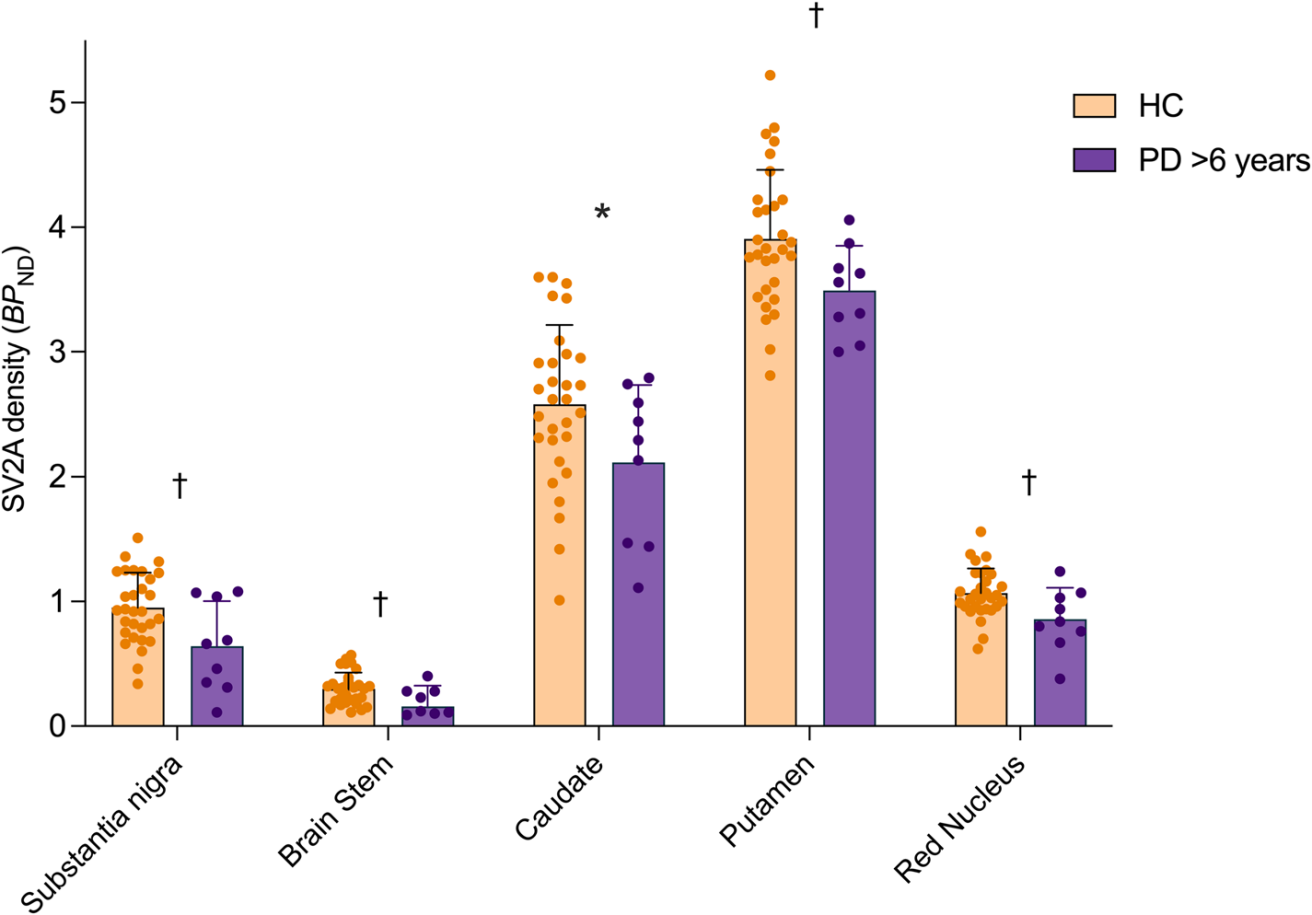
Lower synaptic density (SV2A *BP*_ND_) in ‘longer illness’ PD (>6 yrs) vs HC in primary ROIs. Synaptic density (SV2A *BP*_ND_) across primary (left of dotted line) and secondary (right of dotted line) regions in the longer duration of illness PD subgroup (*n* = 9, purple) vs. HC (*n* = 30, orange) groups. Error bars represent standard deviation. *significant after Bonferroni correction (*p* < 0.01), † significant at *p* < 0.05 (uncorrected) based on post-hoc *t-*tests (two-tailed).

In an exploratory manner, we assessed the extent and distribution of synaptic density loss in a subgroup of the PD cohort with a longer duration (>6 years) of illness. In this longer illness PD subgroup (*n* = 9), we found significantly lower synaptic density compared to HC (*n* = 30) across primary regions (F_1,37_ = 14.2, *p* = 0.0006, Fig. 3). Specifically, post-hoc *t*-tests indicated significantly lower synaptic density with larger differences across all primary regions - substantia nigra (−33%, *p* = 0.036), brainstem (−47%, *p* = 0.039), caudate (−15%, *p* = 0.005), putamen (−10%, *p* = 0.017) and red nucleus (−20%, *p* = 0.042), with effect sizes (Cohen’s *d*), ranging from 0.83 to 1.06. Only the difference in the caudate survived Bonferroni correction (*p* < 0.01) among primary ROIs. In contrast with the full PD cohort, many exploratory regions exhibited significantly lower synaptic density in the longer illness PD subgroup vs. HC (Supplementary Fig. 4, Supplementary Table 3). Out of these, the synaptic loss in the cerebellum, thalamus, parahippocampal area, orbitofrontal and olfactory cortices survived Bonferroni correction (*p* < 0.0024).

### Brain perfusion (R_1_) is altered in PD vs. HC

**Fig. 4:**
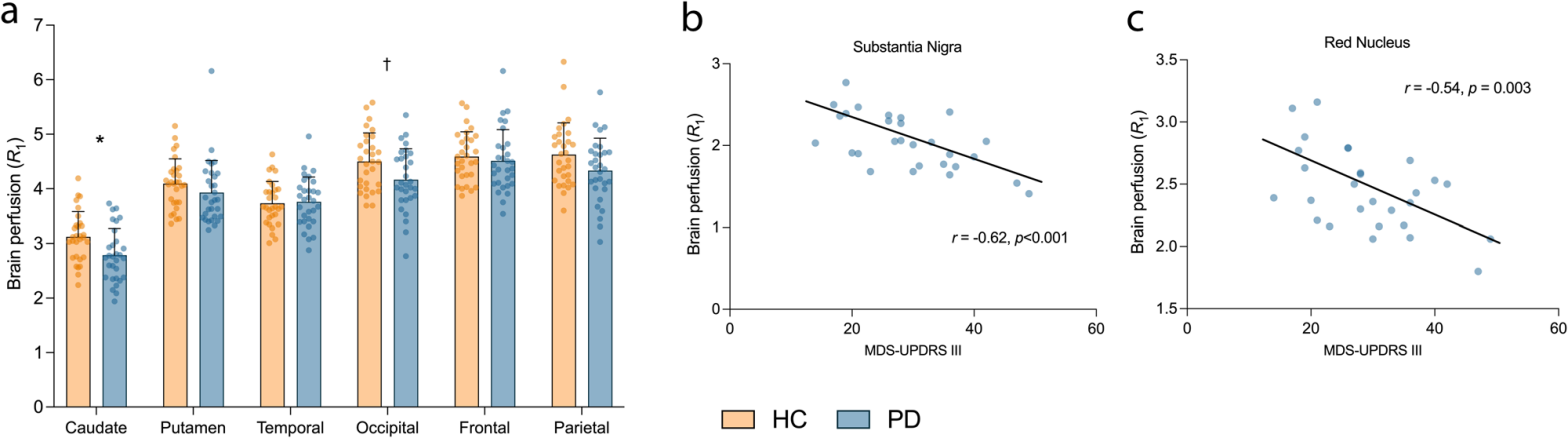
Brain perfusion (*R*_1_) alterations in PD. **a** Relative brain perfusion (*R*_1_) across primary regions in HC (*n* = 30, orange) vs. PD (*n* = 30, blue). Error bars represent standard deviation. *significant after Bonferroni correction (*p* < 0.008), † significant at *p* < 0.05 (uncorrected) based on post-hoc *t-*tests (two-tailed). **b** Correlation between perfusion in the substantia nigra and motor symptom severity (MDS-UPDRS III) in PD patients (*n* = 28) survived Bonferroni correction across all exploratory ROIs (*p* < 0.0025). **c** Correlation between perfusion in the red nucleus in PD patients (*n* = 28) and motor symptom severity (MDS-UPDRS III).

Cortical lobes, caudate and putamen were selected as the *apriori* brain regions to assess perfusion changes using [^11^C]UCB-J *R*_1_ (indicative of brain perfusion). A linear mixed model (see, *Materials and Methods: Statistics*) showed a main effect of diagnosis with significantly lower *R*_1_ (corresponding to lowered perfusion) in PD (*n* = 30) vs. HC (*n* = 30) across the primary *R*_1_ regions (F_1,58_ = 8.08, *p* = 0.006, Fig. 4, Supplementary Table 4). Post-hoc univariate *t*-tests indicated lower *R*_1_ in the caudate (−11%, *p* = 0.0078) and occipital cortex (−7%, *p* = 0.02), with the decrease in parietal cortex being close to significance (−6%, *p* = 0.06). Amongst the primary ROIs, only the group difference in the caudate survived Bonferroni correction (*p* < 0.008). There were no significant differences in secondary regions (Supplementary Table 4). No hemispherical asymmetries were detected in the regional *R*_1_ values in PD subjects.

In the primary ROIs, [^11^C]UCB-J *R*_1_ was significantly negatively-correlated with both motor and total symptom severity in the temporal cortex (*r* = −0.47, *p* = 0.011/*r* = −0.46, *p* = 0.014) and motor severity in the occipital cortex (*r* = −0.38, *p* = 0.049; Supplementary Fig. 5) but these correlations did not survive Bonferroni correction threshold of *p* < 0.01. Of the exploratory regions for *R*_1_, correlations between *R*_1_ and motor/total symptom severity were significant in the substantia nigra (*r* = −0.62, *p* < 0.001/*r* = −0.63, *p* < 0.001) and red nucleus (*r* = −0.54, *p* = 0.003/*r* = −0.52, *p* = 0.005) (Fig. 4). Only the correlation in the substantia nigra was significant after Bonferroni correction (*p* < 0.0025).

**Fig. 5:**
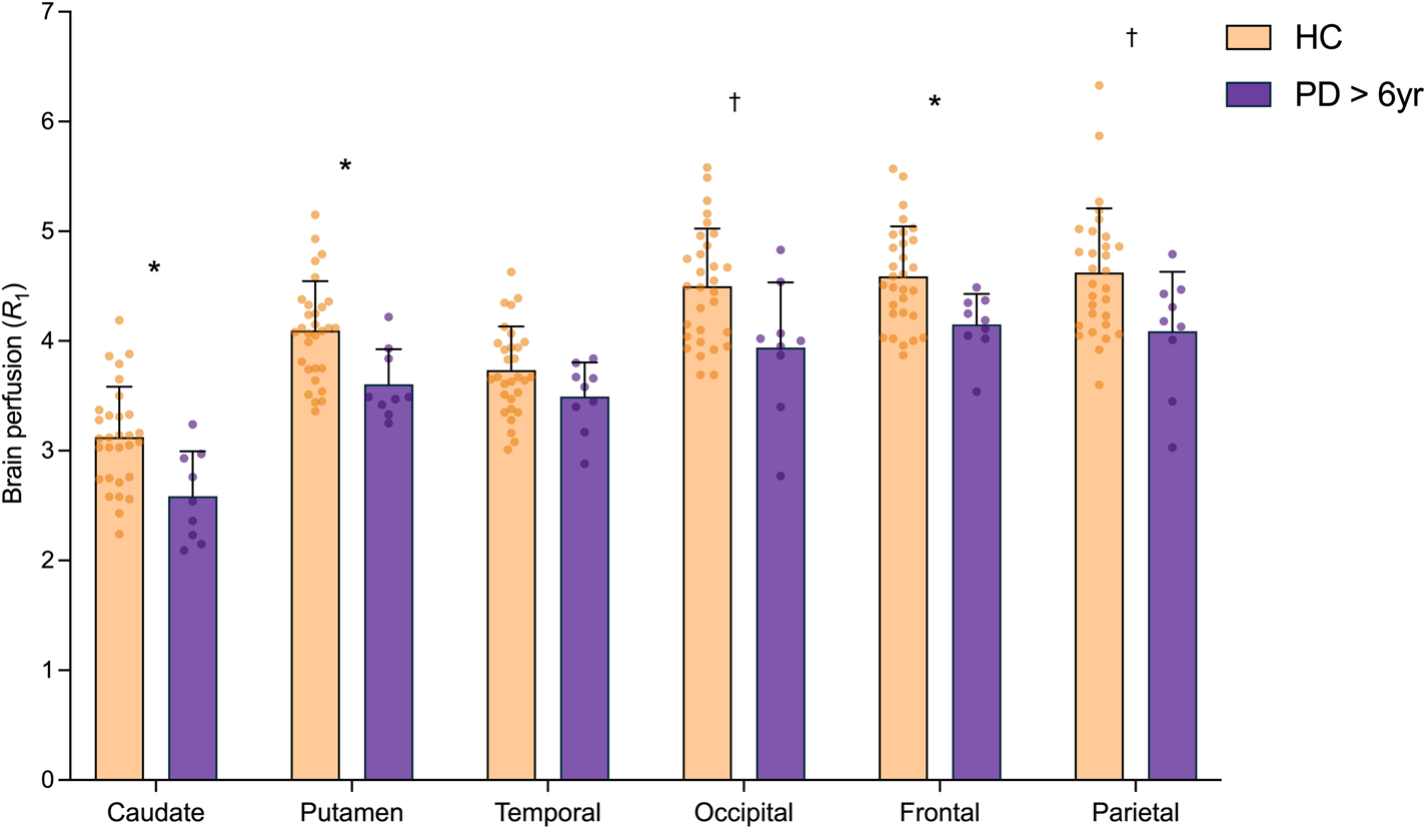
Brain perfusion (*R*_1_) losses in the ‘longer illness’ PD (>6 yrs) subgroup in primary ROIs. Brain perfusion (*R*_1_) across *apriori* selected primary regions in HC (*n* = 30, orange) and PD subgroup with longer illness (*n* = 9, purple). *significant after Bonferroni correction (*p* < 0.008), † significant at *p* < 0.05 (uncorrected) based on post-hoc *t-*tests (two-tailed). Error bars represent standard deviation.

We also examined the extent and pattern of perfusion (*R*_1_) differences in the longer illness PD group. The difference in *R*_1_ between this longer illness group (*n* = 9) and HCs (*n* = 30) was highly significant (F_1,37_ = 11.20, *p* = 0.002). Specifically, *R*_1_ was lower in the caudate (−17%, *p* = 0.004), putamen (−12%, *p* = 0.002), occipital (−12%, *p* = 0.027), parietal (−12%, *p* = 0.023) and frontal (−9%, *p* = 0.002) cortex (Fig. 5). The reductions in perfusion in the caudate, putamen and frontal lobe remained significant after Bonferroni correction (*p* < 0.008). Of the secondary ROIs, *R*_1_ was lower in the red nucleus, supplementary motor cortex, ACC, orbitofrontal and vmPFC cortex, olfactory cortex, thalamus and subthalamic nucleus and ventral striatum (*p* < 0.05, two-tailed; Supplementary Fig. 6, Supplementary Table 5). Of these, only the reductions in the olfactory cortex, orbitofrontal cortex and vmPFC remained significant after Bonferroni correction (*p* < 0.0025).

### Regional perfusion (R_1_) values are associated with cognitive performance in the PD cohort

Montreal cognitive assessment (MoCA) scores were positively associated with *R*_1_ values in some exploratory regions like the cerebellum (*r* = 0.47, *p* = 0.008), pallidum (*r* = 0.42, *p* = 0.02) and raphe nucleus (*r* = 0.38, *p* = 0.04) in PD subjects (Supplementary Fig. 7), but these did not survive Bonferroni correction threshold (*p* < 0.0025). No associations were found between MoCA scores and synaptic density values in any brain region.

### Association between regional synaptic loss and brain perfusion loss in PD

**Fig. 6:**
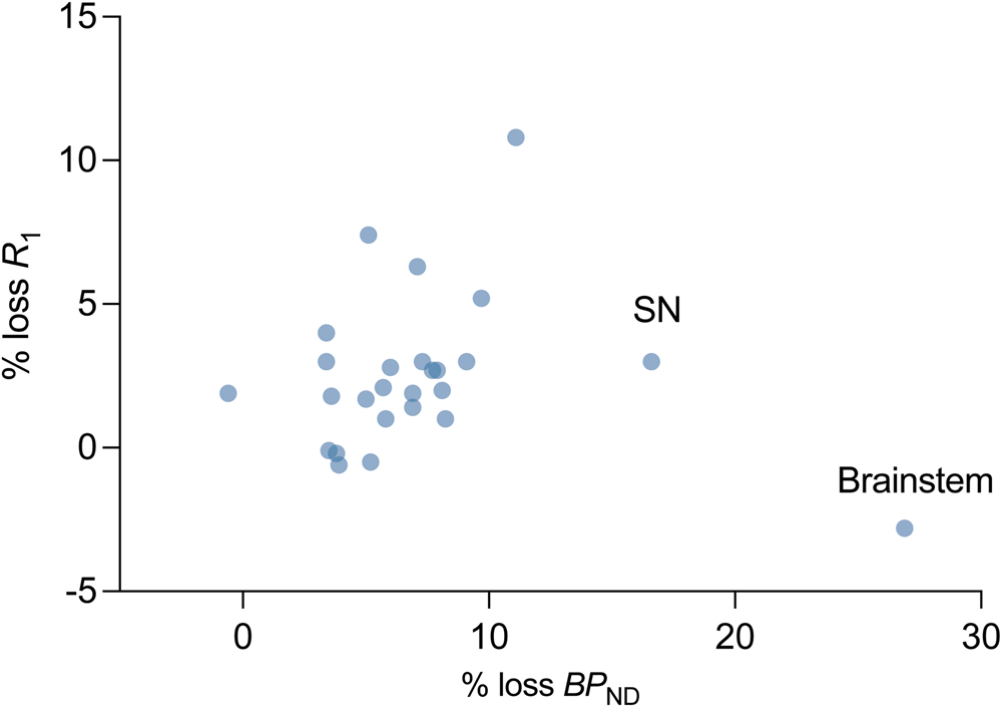
Association between % loss in *BP*_ND_ and *R*_1_ in PD. Scatter plot where each point represents either a primary or a secondary region. Mean % loss in *BP*_ND_ and *R*_1_ (across subjects) for 26 brain regions was computed by comparing it to the respective mean values for HC cohort. Apart from the substantia nigra and brainstem, the losses in *BP*_ND_ and *R*_1_ were correlated significantly (Pearson’s *r* = 0.47, *p* = 0.02).

We computed the average regional percent synaptic (SV2A) loss and percent brain perfusion loss in PD subjects with respect to the mean synaptic density (SV2A *BP*_ND_) and brain perfusion (*R*_1_) in controls for all ROIs (primary and secondary). The relationship between percent synaptic loss and percent perfusion loss was visualized in Fig. 6 using a scatter plot. There was a greater magnitude of average synaptic loss (across subjects and ROIs: 7.2 ± 5.1%), as compared to perfusion loss measured by *R*_1_ (2.5 ± 2.7%). Apart from the brainstem and the substantia nigra, which showed a much higher synaptic loss as compared to brain perfusion, generally synaptic loss showed a positive correlation with brain perfusion loss (Pearson’s *r* after removing brainstem and nigra: 0.47, *p* = 0.02, *n* = 24). The pattern of association between synaptic loss and perfusion loss was similar for the PD cohort with longer illness duration (Supplementary Fig. 8).

## Discussion

We present findings from the largest cross-sectional study of synaptic density imaging in PD to date. Synaptic density was significantly lower in PD compared to matched HCs in regions known to be affected early by PD pathology, including the substantia nigra, brain stem, striatum (caudate) and olfactory cortex. Synaptic density was also correlated with MDS-UPDRS motor scores in key brain regions associated with PD pathology. Specifically, lower synaptic density in substantia nigra was associated with worse motor severity. In a secondary analysis, we found that the longer illness PD subgroup (>6 years since symptom onset) exhibited a greater magnitude and extent of synaptic loss – extending beyond the substantia nigra and brainstem regions to other sub-cortical and cortical regions. Finally, we provide evidence for lowered brain perfusion in PD, in regions that correspond with known metabolic topography of the disease.

Our primary findings are in line with the existing synaptic density imaging literature in PD, which demonstrates the presence of synaptic loss in substantia nigra brainstem regions in vivo^5–9^. Even though the synaptic loss in the substantia nigra did not survive the Bonferroni correction threshold for multiple comparisons, the increased magnitude of synaptic loss (from 17% to 33%) and effect size (Cohen’s *d* from 0.5 to 1) in the longer duration of illness subgroup, along with a significant association between the synaptic density in the nigra and motor severity of the disease in PD patients, indicate that the measured synaptic loss in the nigra is unlikely to be a false positive. We also show evidence of synaptic loss in the olfactory cortex, dorsal striatum (caudate), consistent with striatal function and nigrostriatal circuits being severely impaired in PD^22^. Other synaptic imaging studies have shown non-significantly lower synaptic density in the striatum^5,6,8^. A larger sample size and high-resolution PET imaging likely increased our ability to detect striatal differences. The magnitude of difference was still lower in the striatum than in substantia nigra and brainstem regions - a possible explanation being a smaller proportion of dopaminergic synapses in the striatum, as has been shown in rats^23^.

The synaptic imaging PET studies to date have either focused on early (within 2 years) PD or grouped patients with varying disease duration together. In a secondary analysis, we leveraged the considerable range of disease duration (1–11 years) in our cohort, to focus on a longer illness PD subgroup (>6 years). This subgroup showed a greater magnitude of synaptic loss in the substantia nigra and brain stem regions (effect sizes >0.7) and, importantly, demonstrated synaptic loss beyond these primary regions. Specifically, we observed synaptic loss in cortical regions (frontal lobe ROIs such as olfactory and orbitofrontal cortex was most significantly affected), subcortical regions (thalamus and parahippocampal gyrus) and cerebellum in this subgroup with longer disease duration. These findings are largely in support of Braak’s staging hypothesis, which poses that PD pathology (namely, toxic aggregation of α-synuclein) originates in the brainstem/olfactory system before propagating to the substantia nigra, then subsequently spreading to subcortical and cortical regions as the disease progresses^2^. Indeed, evidence of synaptic loss in the cortex has been demonstrated in both PD dementia and Lewy-body dementia, suggesting that pathology and subsequent synaptic loss spread beyond the substantia nigra as severe cognitive impairment develops^9^.

A recent longitudinal PET study in early PD showed no evidence of progressive synaptic loss over a 2-year period, despite a reduction in dopamine transporter (DAT)^8^. It could be that changes are too subtle for [^11^C]UCB-J to detect within a 2-year period at the sensitivity levels offered by current PET instrumentation, and that a wider window of disease duration or disease stages is needed to detect progressive synaptic loss using PET. Our findings provide support for this, indicating a greater extent and spread of synaptic loss in PD patients with a disease duration greater than 6 years (see Supplementary Figs. 9 and 10 for a comparison between controls and PD cohorts with less than, and greater than, six years of disease duration). There could also be a non-linear trajectory of pathological changes and synaptic loss, such that changes are greater as the disease progresses. Although DAT appears to be a reliable marker of progression in relation to dopaminergic pathology, synaptic imaging with PET provides an important advantage by allowing tracking of the impact of PD pathology on non-dopaminergic systems, as well as in cortical areas. Indeed, while the motor symptoms are well understood in terms of loss of dopaminergic terminals in the nigrostriatal pathway, the mechanisms underpinning non-motor symptoms (e.g., sleep disturbance, depression, constipation and cognitive impairment), which can be as debilitating as the motor symptoms, are less well understood^24^. Synaptic imaging could allow us to tease out distinctions in the pattern of synaptic loss across PD, in relation to both motor and non-motor symptoms. Ultimately this could help inform treatment strategies that are tailored to the pathology, symptomatology and prognosis of distinct PD subtypes. Combining data across synaptic imaging studies in PD could make this endeavor possible.

The utility of a biomarker is often tied to its relationship with symptoms. We observed significant associations between motor severity and synaptic density in substantia nigra, suggesting that synaptic density, measured with PET, is a clinically meaningful marker of PD pathology. One other synaptic imaging study to date has reported an association between synaptic density and PD symptoms, specifically in the lower brainstem^7^. The failure of other PET studies to detect an association between synaptic density and clinical symptoms is likely due to a combination of small sample sizes, a narrow window of disease duration, more limited spatial resolution and heterogeneity in symptoms. Additionally, a consistent medication ‘OFF’ state between clinical evaluations and PET imaging was likely crucial for observing these associations. We observed correlations with motor function specifically in the substantia nigra – the primary pathological site of dopaminergic cell loss - confirming the known pathology and clinical manifestation of PD. A similar correlation was also observed in the red nucleus the red nucleus (but did not survive Bonferroni correction) - a lesser known structure associated with PD - plays an intricate role in coordination of sensorimotor information, relaying information from the motor cortex to the cerebellum^25^, such that this finding may also be relevant. Indeed pathology and synaptic loss in the red nucleus likely contributes to the disturbances in gait seen in PD^26^. Given the regional specificity of these correlations, along with a robust sample size, we can infer with some confidence that synaptic density, as measured with PET, is a clinically meaningful measure in PD.

Another potentially clinically meaningful measure is *R*_1_, a marker of relative brain perfusion which tracks closely with metabolism as measured by [^18^F]FDG PET^12^. We observed significantly lower levels of brain perfusion in PD in a pattern distinct from our SV2A *BP*_ND_ findings - with the largest differences observed in the caudate (survived Bonferroni correction), occipital and parietal regions. The perfusion deficits were higher (with Cohen’s *d* ~ 1 or higher) in the caudate, putamen, frontal, occipital and parietal lobes in longer illness duration PD group with the reductions in the striatum and frontal lobes significant after Bonferroni correction. These findings are partially in line with a robust body of [^18^F]FDG PET work indicating a unique metabolic pattern in PD (the Parkinson’s Disease Related Pattern - PDRP), characterized by hypometabolism in in parietal, occipital and frontal regions, and hypermetabolism in pallido-thalamic regions, midbrain and cerebellum^27^. A recent PET study also demonstrated reduced blood flow in frontal, parietal and occipital regions using the DAT tracer [^18^F]FE-PE2I *R*_1_, which was validated by [^15^O]H_2_O PET^28^. We also found significant correlations between clinical scores (motor and total severity) and *R*_1_ in the substantia nigra (survived Bonferroni correction) and red nucleus, despite observing no between-group difference in *R*_1_ in these regions, suggesting that while the changes are subtle, they are sensitive to disease severity. Further, we did observe an association between cognitive functioning (MoCA) and brain perfusion in the cerebellum, raphe nucleus and pallidum. However, not only did correlations with MoCA not survive correction for multiple comparisons, but our study design was also not suitable for tests regarding cognitive symptoms of PD (subjects with MoCA < 21 were excluded, detailed tests for cognitive and executive function were not performed). Nevertheless, the cerebellum is known to regulate motor and cognitive functions through the cerebello-thalamo-cortical circuit^29^, and the key role of the cerebellum in cognition is being increasingly recognized^30^. Indeed, abnormalities in cerebellar structure and function have been repeatedly observed in PD, including in relation to cognition^31^, such that lower brain perfusion in the cerebellum could be contributing to the cognitive impairment often seen in PD. Future studies with a prospective design to study cognitive deficits in PD should investigate the involvement of the cerebellum.

Our concurrent measurements of synaptic loss and perfusion alterations in PD indicate highest synaptic losses in the brainstem and substantia nigra with relatively lower magnitude of changes in perfusion (Fig. 6). Notably, these regions have been proposed to be amongst the first to be affected by PD pathology in the brain. Other brain regions show a more linear relationship between synaptic and perfusion losses, which provide clues into the structural and functional alterations that occur in PD. Indeed, while *BP*_ND_ and *R*_1_ are measuring fundamentally different things, they are likely linked mechanistically^12^. Understanding the association between *BP*_ND_ and *R*_1_ longitudinally would help to unravel which comes first, and whether this changes as the disease progresses. Further, in our study we observed a higher magnitude of synaptic loss than losses in relative brain perfusion (compared to controls), while a previous study with [^11^C]UCB-J and [^18^F]FDG PET in Parkinson’s disease cohort with and without dementia observed a higher loss in metabolism^32^. This could be an example where glucose metabolism is more sensitive than blood flow but can also be caused due to dissociation between glucose metabolism and blood flow^33^. While further work is needed to validate the use of [^11^C]UCB-J *R*_1_ as a measure of brain perfusion, perhaps against [18F]FDG PET in addition to other flow markers,_,_ these initial findings are in line with the metabolic pattern observed in PD, and imply that [^11^C]UCB-J PET can be used to provide valuable information on both synaptic density and brain perfusion.

There are several limitations to this study. Firstly, all patients were in H&Y stage 2 of the disease. An obvious next step is to examine synaptic density and perfusion across disease stages. Despite this limitation, including only patients in stage 2 allowed us to examine more subtle differences, such as widespread lowering of synaptic density in those with a longer duration of illness, as well as correlations with symptoms. Another limitation is that we cannot be certain that SV2A is solely a marker of synaptic loss and that synaptic vesicle function, for example, is not playing a role^34^. However, given the localization of our findings to the known pathology of PD, and association with symptoms in these regions, we are confident we are primarily measuring synaptic loss. Third, we did not detect any laterality in either synaptic density or brain perfusion. Whereas laterality in striatal regions can often be detected with DAT imaging, [^11^C]UCB-J does not seem to be as sensitive. It will be important to determine whether laterality can be detected in later disease stages. Finally, most patients were taking carbidopa/levodopa, and effects of these medications on synaptic density and brain perfusion cannot be ruled out despite the withholding of medications before the scan.

Important follow-up work includes determining patterns of synaptic loss across disease stages and conducting a longitudinal study beyond two years. Collection of more [^11^C]UCB-J scans, and possible data-sharing across different research groups, can help create sufficient dataset for application of multivariate voxel-based methods to identify networks of synaptic loss in clinically distinct subtypes of PD. Linking synaptic loss to the underlying pathology will also be important; a recent longitudinal multi-tracer study in mild cognitive impairment showed that synaptic loss followed a specific pattern of tau deposition at follow-up^35,36^. Radiotracers for α-synuclein appear to be on the horizon^37^, such that connecting synaptic loss to PD-specific pathology may be possible, hopefully in the near future. Another promising direction is the use of an ultra-high-resolution and sensitivity PET camera (such as the NeuroEXPLORER^38^) to measure synaptic density in small regions such as the substantia nigra and brainstem nuclei with greater accuracy and precision, which should allow detection of earlier and more subtle changes.

To conclude, in the largest cross-sectional synaptic imaging PET study in PD to date, we confirmed previous findings of lower synaptic density in substantia nigra, and extend these findings with evidence of synaptic loss in the brainstem, striatum (caudate), olfactory cortex and other regions in the frontal lobe. Further, we detected correlations in substantia nigra and red nucleus, suggesting that lowered synaptic density in these regions underpins worse motor function in PD. In a secondary analysis we found a greater extent and wider spread pattern of synaptic loss in a subset of patients with a longer duration of illness (>6 years since symptom onset). Although confirmation is needed in a longitudinal study, they provide some support for Braak staging, suggesting that synaptic loss spreads beyond motor regions over time, and that synaptic imaging with PET could be a sensitive marker for tracking α-synuclein induced synaptic loss in PD. We also provide initial evidence that we can extract meaningful information on brain perfusion alterations in PD from *R*_1_ – a measure of brain perfusion. These conclusions establish synaptic imaging as a useful disease progression biomarker that could play a role in the evaluation of critically-needed disease-modifying interventions.

## Methods

### Magnetic resonance imaging

Each participant underwent a high resolution, three-dimensional magnetization prepared rapid acquisition gradient echo (MPRAGE) T1-weighted sequence to exclude structural abnormality and for co-registration with PET images (flip angle = 9°, echo time = 2.44 ms, inversion time = 900 ms, repetition time = 2500 ms, voxel size 1 mm^3^). All MRI scans were acquired on 3-Tesla Siemens Prisma scanner.

### Positron emission tomography

Each participant then underwent a [^11^C]UCB-J PET scan (up to 90 min in length) on the High Resolution Research Tomograph (HRRT, Siemens CTI, Knoxville, TN, USA). [^11^C]UCB-J was synthesized onsite using previously described methods^39^ and administered intravenously as a bolus using an automated infusion pump (Harvard PHD 22/2000, Harvard Apparatus). Before tracer injection, a 6-min transmission measurement was performed for attenuation correction.

Head motion was monitored continuously using the Polaris Vicra optical tracking system (NDI Systems, Waterloo, Ontario, Canada). Data were reconstructed with corrections for attenuation, normalization, scatter, random data, dead time, and motion using the MOLAR algorithm^40^. We have previously shown that calculation of our outcome measure (binding potential, *BP*_ND_, detailed below) can be reliably obtained without the need for arterial blood data in participants with PD^5^ and Alzheimer’s disease^41^.

### Quantitative analysis of PET data

The primary outcome measure was *BP*_ND_, calculated using the simplified reference tissue model (SRTM2)^42^. Parametric images of *BP*_ND_ were generated using the centrum semiovale as a reference region and a fixed *k*_2_′ value (clearance rate constant, *k*_2_, of the reference region). We used a population averaged *k*_2_ of the centrum semiovale calculated using the 1-tissue compartment model based on our previous work (*k*_2_ = 0.031 ± 0.005 [1/min], *n* = 17)^5,43^. The centrum semiovale was defined based on the white matter probability map (SPM12) of HC subjects’ MR images^43^. Additionally, for 18 PD subjects and 26 HC for whom arterial blood sampling was performed, we did not observe any meaningful difference between either the one tissue compartment model (1TCM)-based volume of distribution (*V*_T_, PD: 4.0 ± 0.6 mL/cm^3^, HC: 4.1 ± 0.6 mL/cm^3^, *p* = 0.30, Supplementary Fig. 11) or tracer uptake parameter (*K*_1_, PD: 0.12 ± 0.016 mL min^−1^ cm^−3^, HC: 0.12 ± 0.017 ml min^−1^ cm^−3^, *p* = 0.97, Supplementary Fig. 12) in the reference region between the two groups. We have previously demonstrated that *BP*_ND_ (calculated using SRTM2 and centrum semiovale as reference), is highly correlated to *BP*_ND_ calculated using the 1TCM and arterial sampling in PD and in HC subjects^5^.

Parametric images of the relative delivery rate (*R*_1_) were also generated using SRTM2 and centrum semiovale as reference region. *R*_1_ is proportional to relative blood flow (*R*_1_ = *K*_1_/ *K*_1_’; *K*_1_ = flow × extraction fraction). *R*_1_ generally has lower inter-subject variability than *K*_1_ and is thought of as a surrogate for relative brain perfusion^12^.

Key brain regions affected in early PD^2,44^ were chosen as the primary regions of interest (ROIs) for *BP*_ND_ (brain stem, substantia nigra, red nucleus, caudate and putamen). Secondary (exploratory) regions included the ventral striatum, locus coeruleus, raphe nucleus, pallidum, amygdala, parahippocampal gyrus, thalamus, subthalamic nucleus, cerebellum, olfactory cortex, precentral gyrus, postcentral gyrus, and supplementary motor area, anterior cingulate, poster cingulate, orbitofrontal, ventromedial prefrontal cortex, frontal, temporal, occipital and parietal cortices.

Since [^11^C]UCB-J *R*_1_ has not been explored previously in PD, the primary ROIs - caudate, putamen, temporal, occipital, frontal and parietal lobes - were selected to assess large scale perfusion changes, based partly on [^18^F]FDG hypometabolism literature^14,15^. All other regions above were included in secondary (exploratory) analysis.

ROIs above were taken from the Anatomical Automatic Labeling for SPM2 (AAL) atlas with the exception of hand-drawn templates in the substantia nigra (defined with a dopamine receptor tracer^45,46^), the locus coeruleus (defined with a norepinephrine transporter tracer^47^), and the raphe nucleus (defined with a serotonin transporter tracer^48^). The centrum semiovale was defined based on an average of individual white matter probability maps, optimized to reflect the core of the centrum semiovale^43^. ROIs were applied to the parametric images using the combined transformations from AAL template to PET space according to previous methods^5^. All cortical and striatal regions (caudate, putamen, ventral striatum), and cerebellum, were gray matter segmented using SPM12 (Wellcome Trust Centre for Neuroimaging, London, UK) and corrected for partial volume effects using the Müller-Gartner (MG-PVC) algorithm^49^.

### Statistics

Statistical analysis was performed in R (v 4.2.2) and MATLAB (v2022a). Shapiro-Wilk tests indicated that all outcome measures (*BP*_ND_, *R*_1_) were approximately normally distributed (*p* > 0.05). Group differences in PET measures were investigated using linear mixed models examining the independent and joint effects of diagnosis (between-subjects fixed factor) and ROI (within-subject fixed factor) for all PET outcomes in the primary ROIs. Two different variance-covariance structures (compound symmetry, unstructured) were used to account for within subject correlations (random factor). Akaike Information Criterion (AIC) was used to choose the best fit for the PET outcomes. As analyses based on mixed models involved a combination of regions with varying levels of binding (and flow for *R*_1_) and noise (based on ROI volume), all PET measures were log-transformed before being fit to linear models.

Correlations between PET outcomes in the primary ROIs and clinical measures of disease severity, and between clinical scores and disease duration, were assessed using Pearson’s *r* as a part of the primary analyses. The overall effect of disease duration (defined from symptom onset) on *BP*_ND_ of substantia nigra (a key region affected by PD pathology) was also assessed using Pearson’s *r*. Univariate regional asymmetries in *BP*_ND_ on the contralateral and ipsilateral side (with respect to disease onset side) were explored. Finally, regional association between synaptic loss and perfusion loss in PD (computed with respect to mean HC values) was investigated through a scatter plot.

Following primary analyses, exploratory post-hoc univariate analyses were performed to assess between-group differences in *BP*_ND_ and *R*_1_ in all ROIs and the correlation of these measures to clinical scores. Further post-hoc analyses were performed to investigate group differences between HCs and a longer illness PD sub-group within the main cohort (PD duration >6 y, *n* = 9) using both mixed models and univariate analyses.

Results for primary and exploratory analyses were corrected for multiple comparisons correction (across ROIs) using Bonferroni correction. Results are first presented at the significance level for *p* < 0.05 (two-tailed, uncorrected), followed by a note identifying the tests that survived Bonferroni correction.

### Supplementary information

Supplemental Material

Reporting-Summary

## Data Availability

Most data from this manuscript are available in the article and in its online supplementary material. Further raw data can be shared on request from the corresponding author (Dr. David Matuskey).
