# Author Correction: Synaptic loss and its association with symptom severity in Parkinson’s disease

**DOI:** 10.1038/s41531-024-00674-6

**Published:** 2024-03-12

**Authors:** Sophie E. Holmes, Praveen Honhar, Sule Tinaz, Mika Naganawa, Ansel T. Hilmer, Jean-Dominique Gallezot, Mark Dias, Yanghong Yang, Takuya Toyonaga, Irina Esterlis, Adam Mecca, Christopher Van Dyck, Shannan Henry, Jim Ropchan, Nabeel Nabulsi, Elan D. Louis, Robert Comley, Sjoerd J. Finnema, Richard E. Carson, David Matuskey

**Affiliations:** 1 Department of Psychiatry, Yale School of Medicine, New Haven, CT USA; 2 Department of Neurology, Yale School of Medicine, New Haven, CT USA; 3 Radiology and Biomedical Imaging, Yale School of Medicine, New Haven, CT USA; 4 Department of Biomedical Engineering, Yale School of Engineering and Applied Sciences, Yale University, New Haven, CT USA; 5 Department of Neurology, University of Texas Southwestern Medical Center, New Haven, CT USA; 6 AbbVie, North Chicago, IL USA

**Keywords:** Parkinson's disease, Motor control

Correction to: *npj Parkinson’s Disease* 10.1038/s41531-024-00655-9, published online 24 February 2024

In this article the author name Elan D. Louis was incorrectly written as Elan Louis secondly, in page 7 the word “evaluation” was inadvertently added. The original article has been corrected.

