# Supplementary material for: Synaptic loss and its association with symptom severity in Parkinson’s disease
Source: NPJ Parkinsons Dis. 2024 Feb 24;10:42. doi: 10.1038/s41531-024-00655-9 (PMC10894197; doi:10.1038/s41531-024-00655-9)
Supplement: Supplementary file 1 — Supplemental Material [file 41531_2024_655_MOESM1_ESM.pdf]

Supplementary Information

Supplementary Tables

Supplementary Table 1. PD medication and total levodopa equivalent daily dose (LEDD) for each patient

Subject	PD Medication	LEDD,mg
1	Amantadine; Pramipexole	600
2	CD/LD CR; CD/LD; Selegiline; Mirapex ER	606
3	Ropinirole	80
4	Safinamide	100
5	CD/LD CR; CD/LD; Ropinirole	93
6	CD/LD	735
7	CD/LD; Ropinirole	946
8	CD/LD; Rasagiline	175
9	CD/LD; Rasagiline; Amantadine	337
10	CD/LD; Selegiline	300
11	CD/LD; Amantadine; Rasagiline; Pramipexole	563
12	CD/LD ER; CD/LD; Amantadine; Rasagiline;	468
13	CD/LD; Amantadine; Rasagiline	500
14	CD/LD	75
15	Rasagiline	100
16	CD/LD	25
17	CD/LD; Ropinirole	103
18	No Medication	0
19	Amantadine; Rasagiline	400
20	Rasagiline	100
21	Rasagiline	100
22	Amantadine; Rasagiline	300
23	CD/LD; Safinamide	
24	Rasagiline	100
25	CD/LD	75
26	CD/LD	75
27	CD/LD	75
28	CD/LD ER; CD/LD	65

29	CD/LD; Selegiline	288
30	CD/LD	50
31	CD/LD	75

CD/LD: Carbidopa/levodopa, CR: Sustained release, ER: Extended release.
LEDD calculated using the Tomlinson formula.

Supplementary Table 2. BP_{ND} values across HC and PD groups. Reported values are Mean (SD).

Region	HC group (n=30)	PD group (n=30)	*p*-value	% Difference	Cohen's d magnitude
Primary					
Substantia nigra	0.95 (0.28)	0.79 (0.30)	0.039	-16.6	0.54
Brainstem	0.30 (0.13)	0.22 (0.14)	0.024	-26.9	0.60
Red nucleus	1.07 (0.20)	0.98 (0.26)	0.147	-8.23	0.38
**Caudate**	**4.18 (0.63)**	**3.72 (0.64)**	**0.006**	**-11.1**	**0.74**
Putamen	5.73 (0.70)	5.53 (0.86)	0.336	-3.4	0.25
Secondary					
Temporal	5.51 (0.76)	5.30 (0.76)	0.278	-3.9	0.28
Occipital	5.68 (0.75)	5.39 (0.81)	0.157	-5.1	0.37
Frontal	5.62 (0.76)	5.34 (0.75)	0.154	-5.0	0.37
Parietal	6.02 (0.86)	5.59 (0.84)	0.055	-7.1	0.51
Cerebellum	3.09 (0.44)	2.93 (0.47)	0.181	-5.2	0.35
Precentral	5.25 (0.74)	5.05 (0.74)	0.299	-3.8	0.27
Postcentral	5.61 (0.84)	5.41 (0.77)	0.340	-3.6	0.25
Supplementary motor	5.19 (0.73)	4.89 (0.68)	0.108	-5.8	0.42
Anterior cingulum	5.06 (0.72)	4.71 (0.70)	0.060	-6.9	0.50
Posterior cingulum	4.03 (0.65)	3.79 (0.70)	0.173	-6.0	0.36
Orbitofrontal	5.45 (0.73)	5.03 (0.72)	0.029	-7.7	0.58
vmPFC	5.47 (0.81)	5.07 (0.73)	0.049	-7.3	0.52
Ventral striatum	5.10 (0.58)	4.93 (0.81)	0.349	-3.4	0.24
Thalamus	2.31 (0.39)	2.12 (0.42)	0.081	-8.1	0.46
Subthalamic	1.20 (0.20)	1.10 (0.25)	0.112	-7.9	0.42
Parahippocampal	3.91 (0.77)	3.56 (0.56)	0.046	-9.1	0.53
Pallidum	2.11 (0.44)	1.99 (0.33)	0.235	-5.7	0.31
Amygdala	3.34 (0.55)	3.11 (0.62)	0.133	-6.9	0.39
Olfactory	4.45 (0.76)	4.02 (0.59)	0.017	-9.7	0.64
Locus coeruleus	0.93 (0.20)	0.93 (0.26)	0.925	0.6	0.02
Raphe	1.13 (0.29)	1.09 (0.37)	0.648	-3.5	0.12

Values in **bold** imply significance after Bonferroni correction

Supplementary Table 3. BP_{ND} values across HC and 'longer illness' (>6 yr) PD group. Reported values are Mean (SD).

Region	HC group (n=30)	>6 yr PD group (n=9)	*p*-value	% Difference	Cohen's d magnitude
Primary					
Substantia nigra	0.95 (0.28)	0.64 (0.36)	0.036	-32.8	1.04
Brainstem	0.30 (0.13)	0.16 (0.16)	0.039	-47.0	1.01
Red nucleus	1.07 (0.20)	0.86 (0.25)	0.042	-19.6	1.00
**Caudate**	**4.18 (0.63)**	**3.55 (0.48)**	**0.005**	**-15.2**	**1.06**
Putamen	5.73 (0.70)	5.18 (0.50)	0.017	-9.6	0.83
Secondary					
Temporal	5.51 (0.76)	4.98 (0.58)	0.038	-9.7	0.74
Occipital	5.68 (0.75)	5.21 (0.72)	0.110	-8.3	0.63
Frontal	5.62 (0.76)	5.05 (0.54)	0.020	-10.0	0.79
Parietal	6.02 (0.86)	5.52 (0.72)	0.100	-8.4	0.61
**Cerebellum**	**3.09 (0.44)**	**2.78 (0.13)**	**0.002**	**-9.9**	**0.78**
Precentral	5.25 (0.74)	4.81 (0.53)	0.060	-8.5	0.63
Postcentral	5.61 (0.84)	5.22 (0.57)	0.129	-6.9	0.49
Supplementary motor	5.19 (0.73)	4.63 (0.53)	0.021	-10.8	0.80
Anterior cingulum	5.06 (0.72)	4.41 (0.56)	0.012	-12.8	0.94
Posterior cingulum	4.03 (0.65)	3.59 (0.42)	0.026	-10.9	0.73
**Orbitofrontal**	**5.45 (0.73)**	**4.74 (0.46)**	**0.002**	**-13.1**	**1.04**
vmPFC	5.47 (0.81)	4.75 (0.52)	0.005	-13.1	0.95
Ventral striatum	5.10 (0.58)	4.54 (0.52)	0.020	-11.0	0.98
**Thalamus**	**2.31 (0.39)**	**1.93 (0.23)**	**0.002**	**-16.2**	**1.03**
Subthalamic	1.20 (0.20)	0.97 (0.31)	0.066	-19.0	0.99
**Parahippocampal**	**3.91 (0.77)**	**3.32 (0.26)**	**0.001**	**-15.0**	**0.86**
Pallidum	2.11 (0.44)	2.02 (0.37)	0.542	-4.4	0.22
Amygdala	3.34 (0.55)	2.82 (0.36)	0.003	-15.6	1.02
**Olfactory**	**4.45 (0.76)**	**3.78 (0.37)**	**0.001**	**-15.1**	**0.97**
Locus coeruleus	0.93 (0.20)	0.89 (0.19)	0.653	-3.5	0.17
Raphe	1.13 (0.29)	0.92 (0.27)	0.063	-18.7	0.75

Values in **bold** imply significance after Bonferroni correction

Supplementary Table 4. R_1 values across HC and PD groups. Reported values are Mean (SD).

Region	HC group (n=30)	PD group (n=30)	*p*-value	% Difference	Cohen's d magnitude
Primary					
**Caudate**	**3.12 (0.46)**	**2.79 (0.49)**	**0.008**	**-10.8**	**0.71**
Putamen	4.09 (0.45)	3.93 (0.59)	0.233	-4.0	0.31
Temporal	3.73 (0.40)	3.76 (0.45)	0.828	0.6	0.06
Occipital	4.50 (0.53)	4.17 (0.57)	**0.022**	-7.4	0.61
Frontal	4.59 (0.46)	4.51 (0.57)	0.573	-1.7	0.15
Parietal	4.63 (0.58)	4.33 (0.59)	0.059	-6.3	0.50
Secondary					
Substantia nigra	2.15 (0.30)	2.08 (0.35)	0.443	-3.0	0.20
Brainstem	1.32 (0.22)	1.36 (0.18)	0.482	2.8	0.18
Red nucleus	2.52 (0.29)	2.50 (0.35)	0.768	-1.0	0.08
Cerebellum	3.29 (0.43)	3.31 (0.40)	0.868	0.5	0.04
Precentral	4.89 (0.55)	4.90 (0.57)	0.960	0.2	0.01
Postcentral	4.73 (0.55)	4.65 (0.56)	0.558	-1.8	0.15
Supplementary motor	4.44 (0.41)	4.40 (0.61)	0.739	-1.0	0.09
Anterior cingulum	3.45 (0.33)	3.40 (0.45)	0.647	-1.4	0.12
Posterior cingulum	3.92 (0.41)	3.81 (0.51)	0.355	-2.8	0.24
Orbitofrontal	4.18 (0.42)	4.07 (0.53)	0.374	-2.7	0.23
vmPFC	4.08 (0.44)	3.96 (0.53)	0.328	-3.0	0.25
Ventral striatum	3.35 (0.34)	3.25 (0.44)	0.324	-3.0	0.26
Thalamus	3.11 (0.36)	3.05 (0.44)	0.554	-2.0	0.15
Subthalamic	2.75 (0.28)	2.67 (0.38)	0.388	-2.7	0.22
Parahippocampal	2.52 (0.31)	2.44 (0.21)	0.280	-3.0	0.28
Pallidum	2.32 (0.22)	2.27 (0.26)	0.427	-2.1	0.21
Amygdala	2.06 (0.23)	2.03 (0.25)	0.534	-1.9	0.16
Olfactory	3.00 (0.33)	2.84 (0.36)	0.089	-5.2	0.45
Locus coeruleus	2.16 (0.34)	2.12 (0.39)	0.675	-1.9	0.11
Raphe	2.38 (0.28)	2.38 (0.34)	0.966	0.1	0.01

Values in **bold** imply significance after Bonferroni correction

Supplementary Table 5. R_1 values across HC and 'longer illness (>6 yr) PD group. Reported values are Mean (SD).

Region	HC group (n=30)	>6 yr PD group (n=9)	p-value	% Difference	Cohen's d magnitude
Primary					
**Caudate**	**3.12 (0.46)**	**2.58 (0.41)**	**0.004**	**-17.3**	**1.20**
**Putamen**	**4.09 (0.45)**	**3.61 (0.32)**	**0.002**	**-11.9**	**1.14**
Temporal	3.73 (0.40)	3.49 (0.31)	0.076	-6.4	0.63
Occipital	4.50 (0.53)	3.94 (0.60)	0.027	-12.4	1.03
**Frontal**	**4.59 (0.46)**	**4.15 (0.28)**	**0.002**	**-9.5**	**1.03**
Parietal	4.63 (0.58)	4.09 (0.54)	0.023	-11.6	0.93

Secondary					
Substantia nigra	2.15 (0.30)	1.90 (0.40)	0.114	-11.65	0.77
Brainstem	1.32 (0.22)	1.29 (0.18)	0.704	-2.09	0.13
Red nucleus	2.52 (0.29)	2.27 (0.31)	0.048	-10.17	0.87
Cerebellum	3.29 (0.43)	3.18 (0.23)	0.333	-3.30	0.27
Precentral	4.89 (0.55)	4.65 (0.32)	0.106	-5.04	0.48
Postcentral	4.73 (0.55)	4.46 (0.43)	0.144	-5.68	0.51
Supplementary motor	4.44 (0.41)	4.11 (0.25)	0.007	-7.56	0.87
Anterior cingulum	3.45 (0.33)	3.13 (0.23)	0.004	-9.25	1.02
Posterior cingulum	3.92 (0.41)	3.55 (0.49)	0.060	-9.53	0.87
**Orbitofrontal**	**4.18 (0.42)**	**3.76 (0.27)**	**0.002**	**-10.10**	**1.07**
**vmPFC**	**4.08 (0.44)**	**3.59 (0.23)**	**0.000**	**-12.24**	**1.24**
Ventral striatum	3.35 (0.34)	3.00 (0.25)	0.003	-10.62	1.10
Thalamus	3.11 (0.36)	2.86 (0.29)	0.050	-7.98	0.72
Subthalamic	2.75 (0.28)	2.41 (0.41)	0.042	-12.37	1.09
Parahippocampal	2.52 (0.31)	2.36 (0.15)	0.054	-6.02	0.53
Pallidum	2.32 (0.22)	2.21 (0.21)	0.211	-4.49	0.48
Amygdala	2.06 (0.23)	1.92 (0.23)	0.115	-7.10	0.65
**Olfactory**	**3.00 (0.33)**	**2.68 (0.15)**	**0.001**	**-10.37**	**1.02**
Locus coeruleus	2.16 (0.34)	2.11 (0.35)	0.746	-2.01	0.13
Raphe	2.38 (0.28)	2.35 (0.32)	0.823	-1.13	0.09

Values in **bold** imply significance after Bonferroni correction

Supplementary Figures

Supplementary Figure 1. Correlation between SV2A density and total MDS-UPDRS score (n=28)

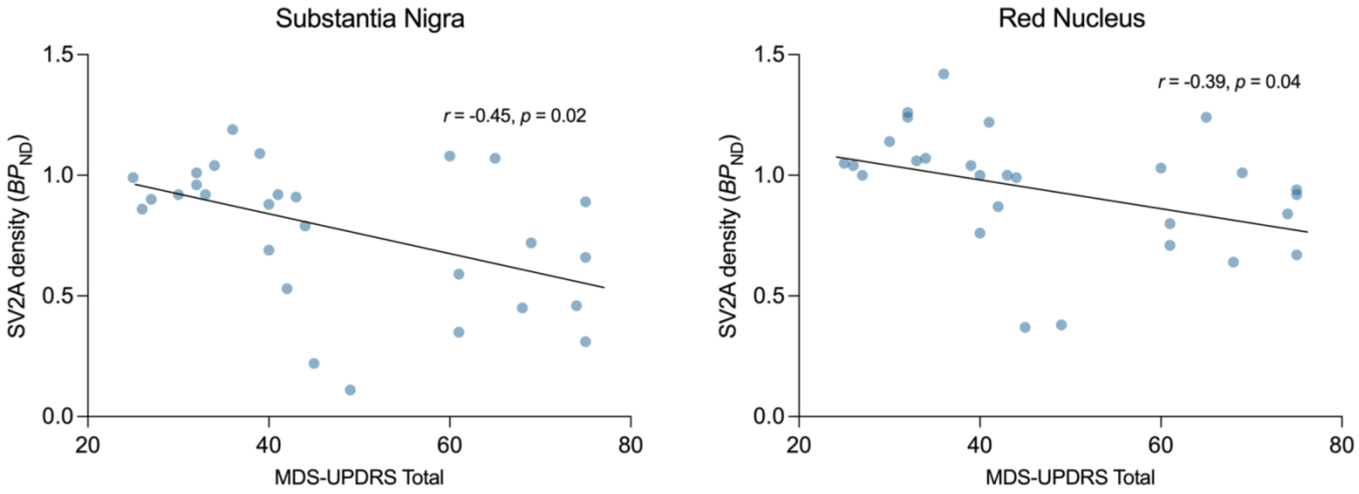

Supplementary Figure 2. Correlation between disease duration and symptom severity (n=31*)

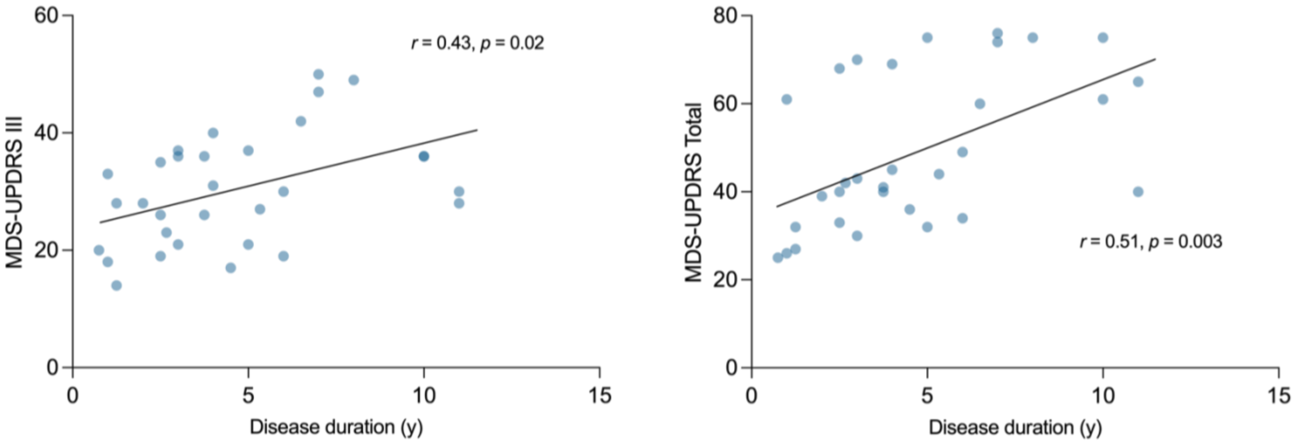

* One subject included here did not have a $[^{11}C]$UCB-J PET scan

Supplementary Figure 3. Correlation between disease duration and substantia nigra BP_{ND} (n=30)

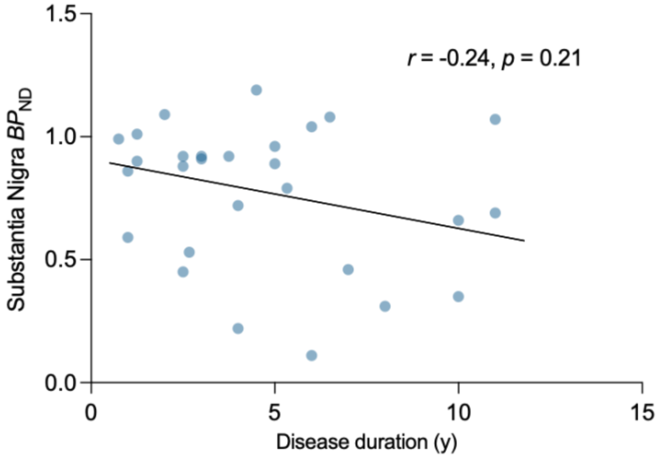

Non-significant negative association between disease duration and synaptic (SV2A) density in the substantia nigra.

Supplementary Figure 4. Synaptic Density (BP_{ND}) across HC (n=30)and 'longer illness' PD (>6 yr) group (n=9)(Secondary ROIs)

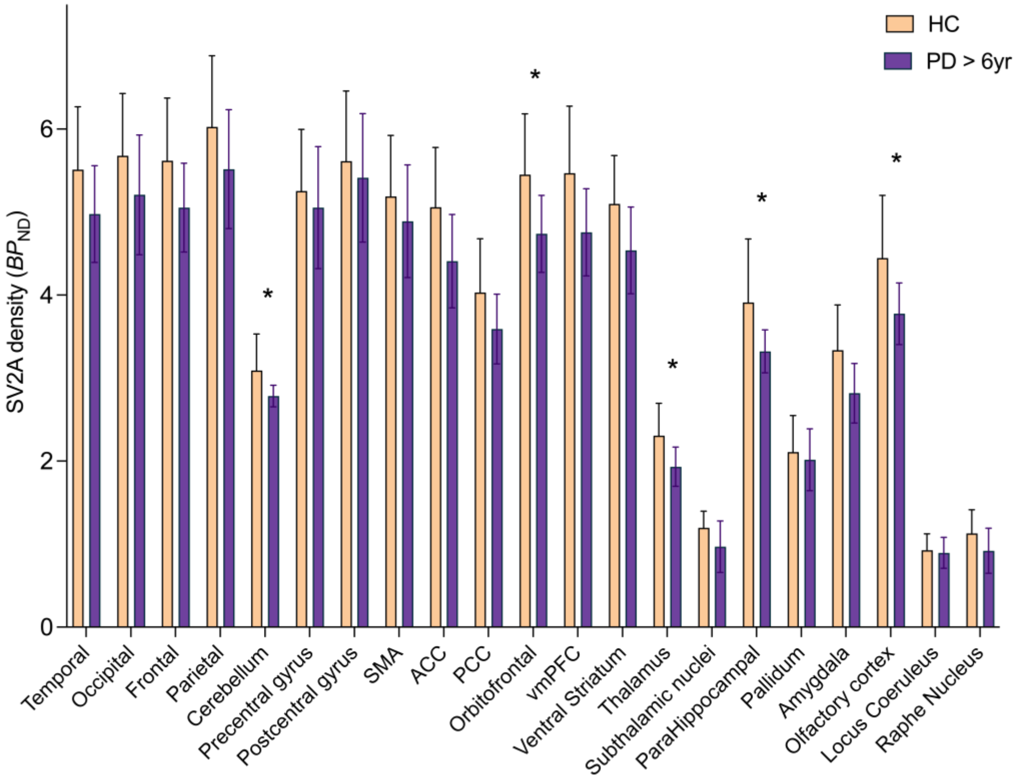

* Group differences that survived Bonferroni Correction ($p < 0.0024$)

Supplementary Figure 5. Correlation between R_1 and symptom severity (n=28)

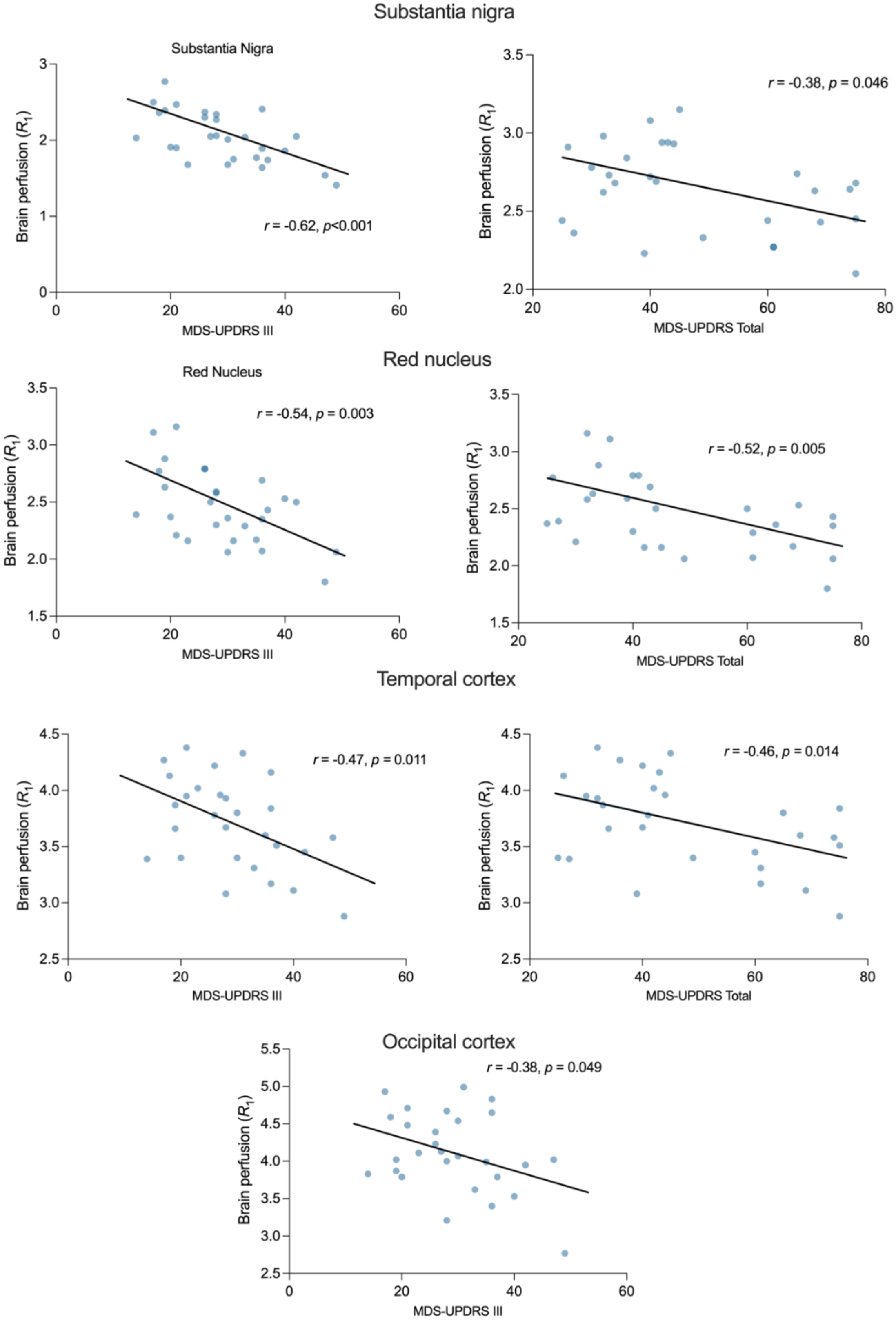

Supplementary Figure 6. Brain Perfusion (*R_1*) across HC (n=30) and 'longer illness' PD (>6 yr) group (n=30) in Secondary ROIs

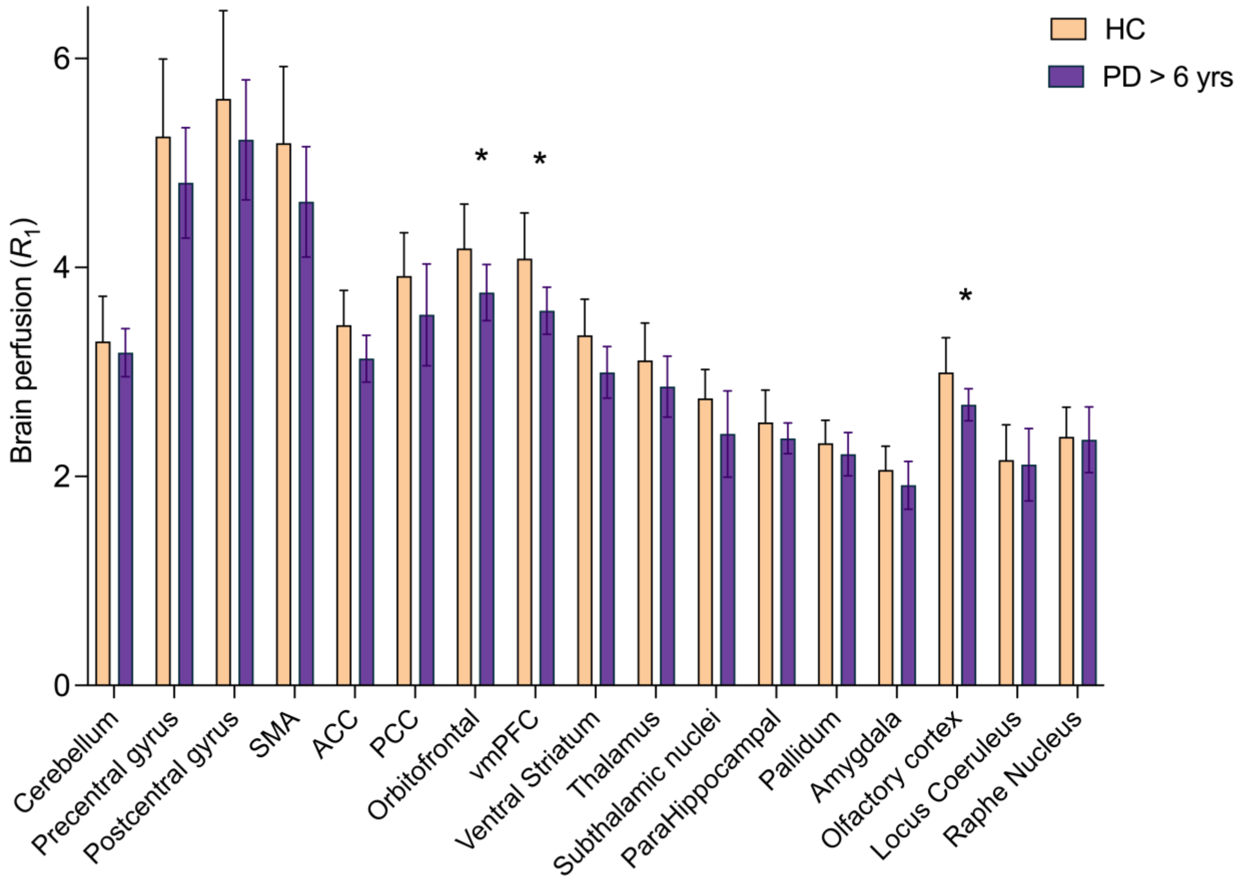

* Group differences that survived Bonferroni Correction ($p < 0.0024$)

Supplementary Figure 7. Correlation between R_1 and MoCA scores (n=30)
(Note none of these correlations survived Bonferroni correction and should be interpreted with caution)

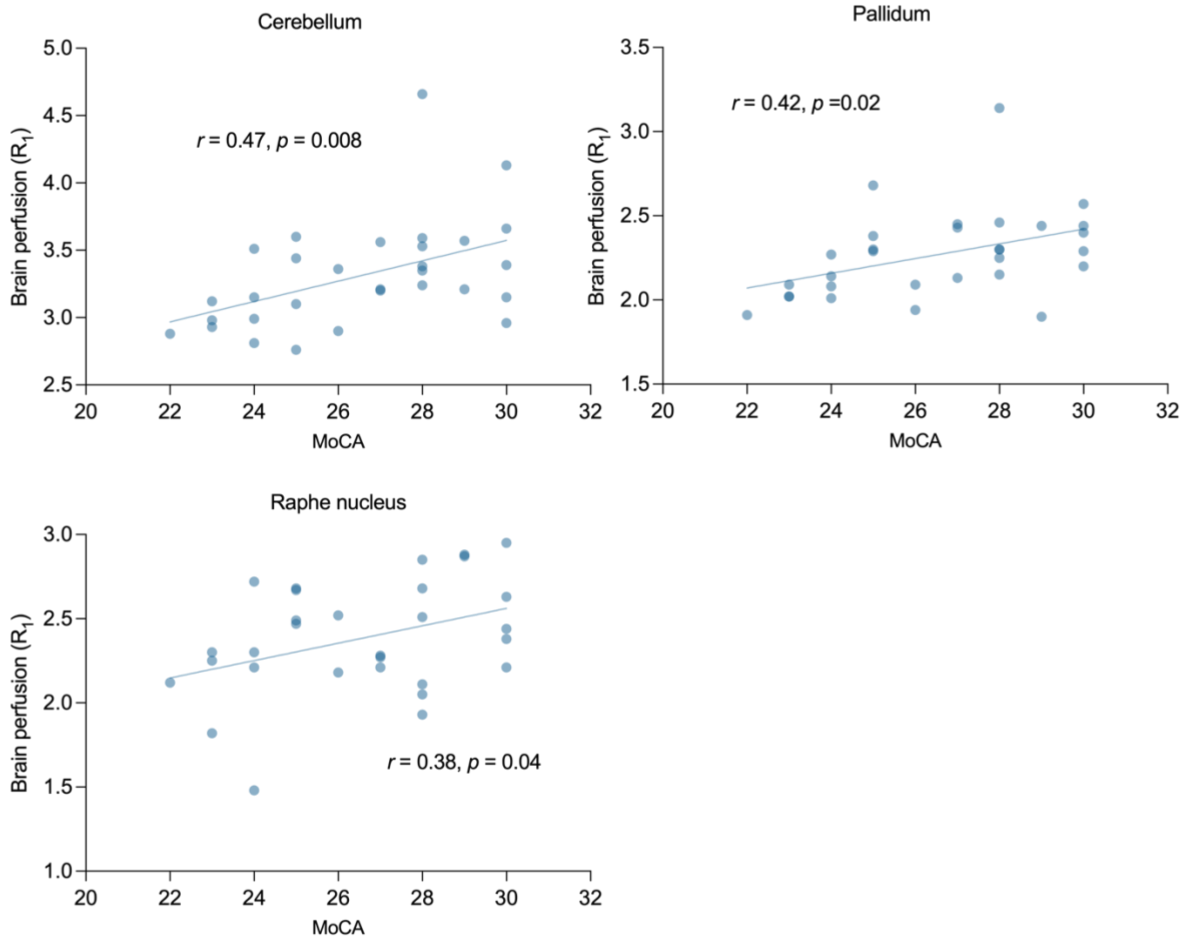

Supplementary Figure 8. Association between % loss in *R_1* and *BP_{ND}* in the 'longer illness' PD group

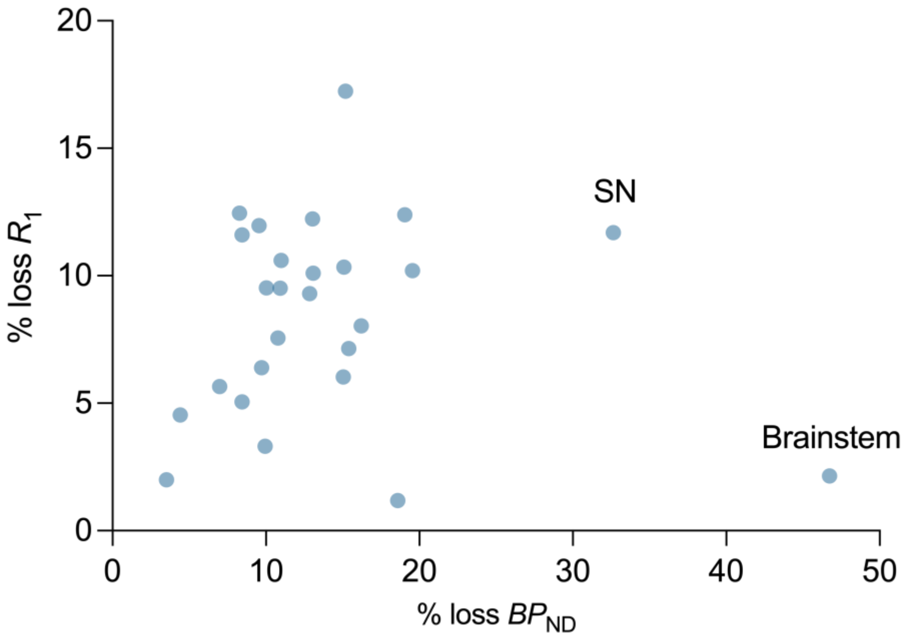

Regional percent losses in R_1 and BP_{ND}. The losses were calculated relative to age and sex matched healthy controls. Each point represents a different brain region, losses were averaged across all Parkinson's subjects. For most brain regions, there is a linear relationship between losses in perfusion and synaptic density, but brainstem and substantia nigra have a much higher magnitude of synaptic loss, compared to perfusion.

Supplementary Figure 9. ***BP_{ND}*** **values across HC and PD subgroups**

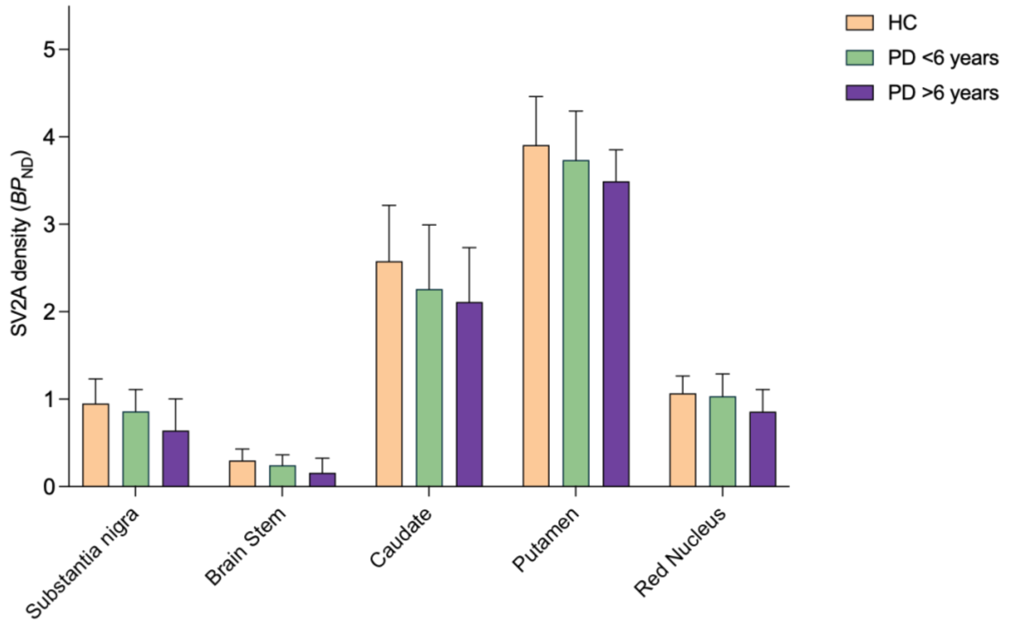

Supplementary Figure 10. ***R_1*** **values across HC and PD subgroups**

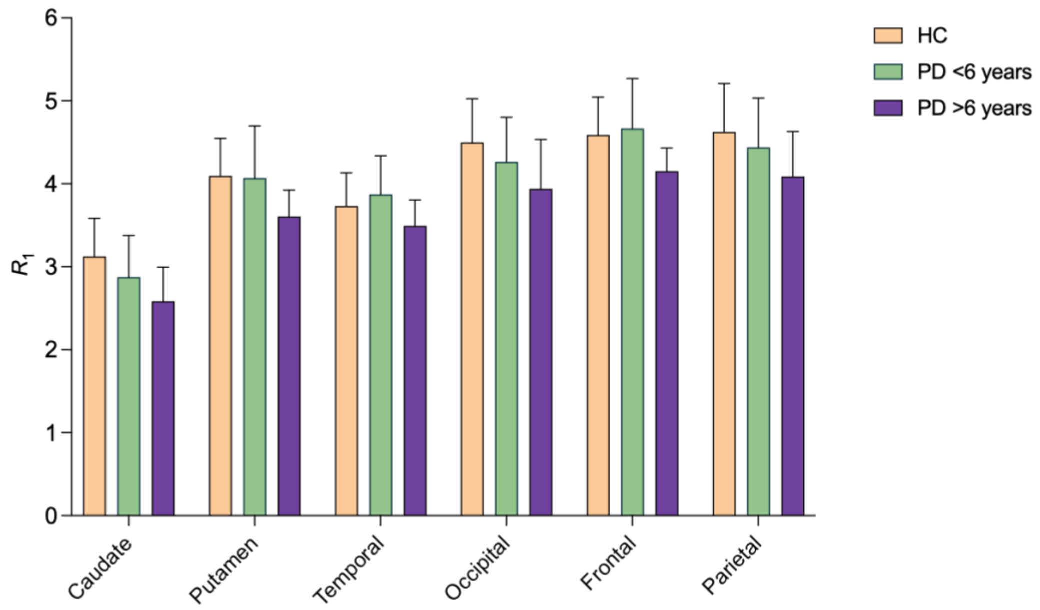

Supplementary Figure 11: Boxplot comparing V_T values in the reference region (Centrum Semiovale) between HC and PD

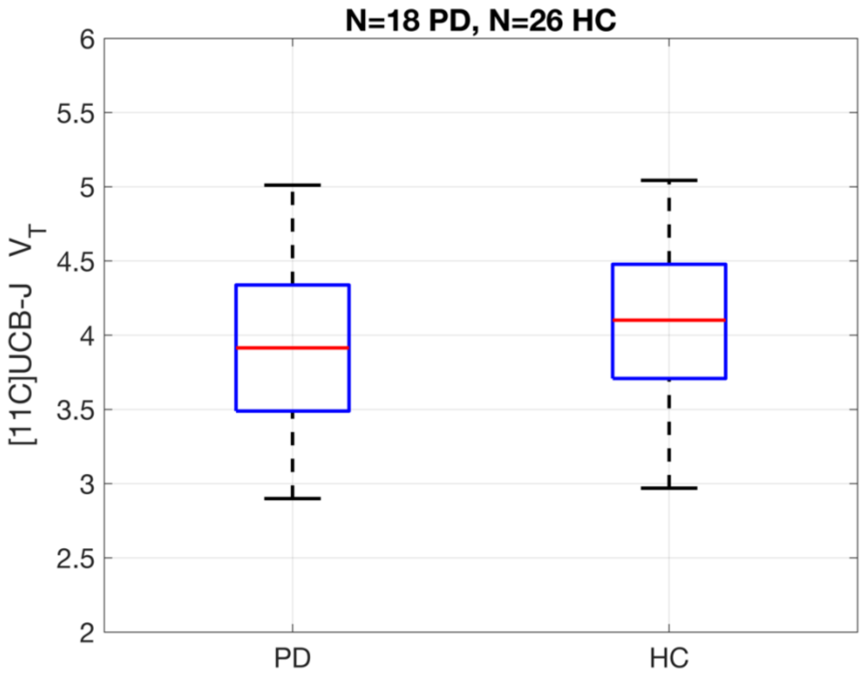

No statistically meaningful difference was observed between V_T values in the centrum semiovale of the two groups. V_T values were computed based on the 1TCM (60 min PET data) in subjects for whom arterial sampling was performed (*n*=18 PD, *n*=26 HC).

Supplementary Figure 12: Boxplot comparing K_1 values in the reference region (Centrum Semiovale) between HC and PD

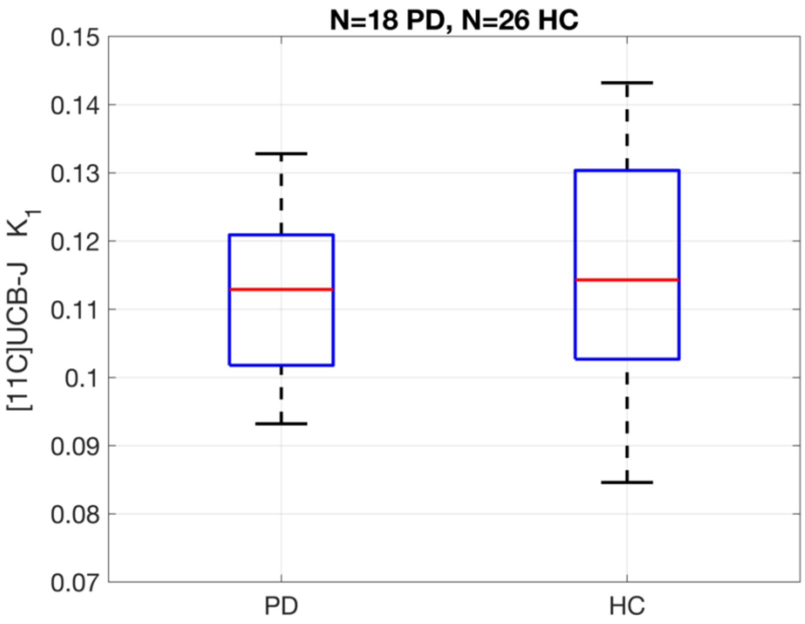

No statistically meaningful difference was observed between K_1 values in the centrum semiovale of the two groups. K_1 values were computed based on the 1TCM (60 min PET data) in subjects for whom arterial sampling was performed (n=18 PD, n=26 HC).

Supplementary Figure 13. Correlations between motor severity and BP_{ND} <6 yr illness duration PD group

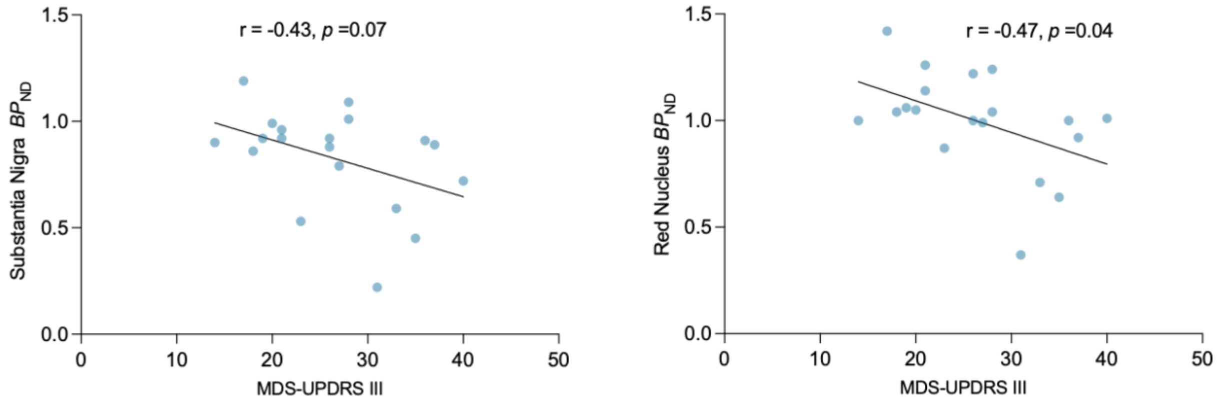

Associations between MDS-UPDRS Part III (motor severity) and synaptic (SV2A) density in the nigra and red nucleus in the PD sub-group with disease duration < 6 yr are similar to the complete PD cohort, with somewhat higher p-values due to the smaller sample (n=21) compared to the complete PD cohort (n=30).

Supplementary Figure 14. Correlations between motor severity and BP_{ND} for >6 yr illness duration PD group

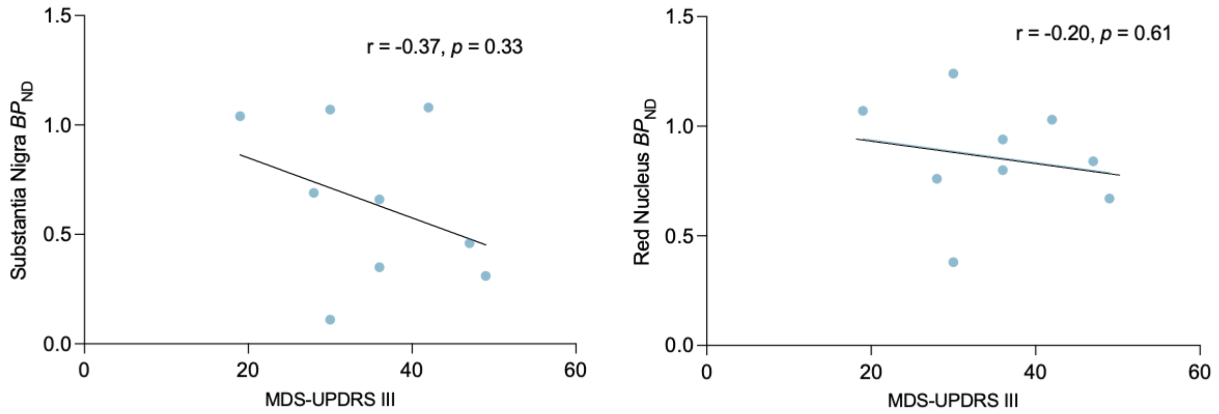

Associations between MDS-UPDRS Part III (motor severity) and synaptic (SV2A) density in the nigra and red nucleus in the PD sub-group with disease duration > 6 yr were smaller compared with the complete PD cohort. However, this might be due to the considerably smaller sample (n=9) compared to the complete PD cohort (n=30).
