# Supplementary material for: Synaptic loss and its association with symptom severity in Parkinson’s disease
Source: NPJ Parkinsons Dis. 2024 Feb 24;10:42. doi: 10.1038/s41531-024-00655-9 (PMC10894197; doi:10.1038/s41531-024-00655-9)
Supplement: Supplementary file 2 — Reporting-Summary [file 41531_2024_655_MOESM2_ESM.pdf]

nature portfolio

Corresponding author(s): David Matuskey

Last updated by author(s): Dec 14, 2023

Reporting Summary

Nature Portfolio wishes to improve the reproducibility of the work that we publish. This form provides structure for consistency and transparency in reporting. For further information on Nature Portfolio policies, see our Editorial Policies and the Editorial Policy Checklist.

Statistics

For all statistical analyses, confirm that the following items are present in the figure legend, table legend, main text, or Methods section.

n/a	Confirmed	
☐	☒	The exact sample size (*n*) for each experimental group/condition, given as a discrete number and unit of measurement
☐	☒	A statement on whether measurements were taken from distinct samples or whether the same sample was measured repeatedly
☐	☒	The statistical test(s) used AND whether they are one- or two-sided<br>*Only common tests should be described solely by name; describe more complex techniques in the Methods section.*
☐	☒	A description of all covariates tested
☐	☒	A description of any assumptions or corrections, such as tests of normality and adjustment for multiple comparisons
☐	☒	A full description of the statistical parameters including central tendency (e.g. means) or other basic estimates (e.g. regression coefficient) AND variation (e.g. standard deviation) or associated estimates of uncertainty (e.g. confidence intervals)
☐	☒	For null hypothesis testing, the test statistic (e.g. *F*, *t*, *r*) with confidence intervals, effect sizes, degrees of freedom and *P* value noted<br>*Give P values as exact values whenever suitable.*
☒	☐	For Bayesian analysis, information on the choice of priors and Markov chain Monte Carlo settings
☒	☐	For hierarchical and complex designs, identification of the appropriate level for tests and full reporting of outcomes
☐	☒	Estimates of effect sizes (e.g. Cohen's *d*, Pearson's *r*), indicating how they were calculated

Our web collection on statistics for biologists contains articles on many of the points above.

Software and code

Policy information about availability of computer code

Data collection	No software was used
Data analysis	Data analysis and statistics was performed using R (v4.2.2) and MATLAB (v2022a). Linear mixed effects modeling was performed using the nlme library in R.

For manuscripts utilizing custom algorithms or software that are central to the research but not yet described in published literature, software must be made available to editors and reviewers. We strongly encourage code deposition in a community repository (e.g. GitHub). See the Nature Portfolio guidelines for submitting code & software for further information.

Data

Policy information about availability of data

All manuscripts must include a data availability statement. This statement should provide the following information, where applicable:

- Accession codes, unique identifiers, or web links for publicly available datasets
- A description of any restrictions on data availability
- For clinical datasets or third party data, please ensure that the statement adheres to our policy

Most of the data required to replicate our results are already available in the Supplementary Information. Data can be assessed with a request to the corresponding author (Dr. David Matuskey).

Research involving human participants, their data, or biological material

Policy information about studies with human participants or human data. See also policy information about sex, gender (identity/presentation), and sexual orientation and race, ethnicity and racism.

Reporting on sex and gender	This work did not consider the effects of sex and gender since we were underpowered to look at those effects in the context of synaptic loss in Parkinson's disease. Instead our patient population was compared to a control cohort that was balanced for sex, such that our findings apply to both sexes.
Reporting on race, ethnicity, or other socially relevant groupings	This work did not consider the effects of race or ethnicity since we were underpowered to look at those effects in the context of synaptic loss in Parkinson's disease.
Population characteristics	Our study population included 30 participants with Parkinson's disease (Hoehn-Yahr Stage 2) and 30 age- and sex-matched controls.
Recruitment	Participants were recruited through a variety of avenues including posting flyers at numerous clinics within the Yale New Haven and Hartford Healthcare networks, as well as community centres. The study was also advertised online, for example through Facebook groups, foundation and support group websites across Connecticut. No biases in recruitment methods are noted and demographics are representative of the geographic area and the patient population.
Ethics oversight	Yale University Human Investigation Committee, Radioactive Drug Research Committee

Note that full information on the approval of the study protocol must also be provided in the manuscript.

Field-specific reporting

Please select the one below that is the best fit for your research. If you are not sure, read the appropriate sections before making your selection.

☒ Life sciences ☐ Behavioural & social sciences ☐ Ecological, evolutionary & environmental sciences

For a reference copy of the document with all sections, see nature.com/documents/nr-reporting-summary-flat.pdf

Life sciences study design

All studies must disclose on these points even when the disclosure is negative.

Sample size	The current sample size represents the largest cross-sectional study with synaptic density PET in Parkinson's (60 total participants). Previous studies have already shown significant measurements at smaller sample sizes, this larger study was partly aimed at confirming those findings.
Data exclusions	No data were excluded. Clinical motor exam scores were unavailable for 2 out of 30 Parkinson's disease participants.
Replication	The main research tool in this study was PET imaging. PET imaging results in radioactive exposure for human participants. Due to this only one PET scan was acquired for each participant (as per ALARA principles of radiation). However, test-retest replication validation has already been performed for this technique in the past.
Randomization	To assess synaptic changes in the brain due to Parkinson's disease, we compared the cohort of 30 Parkinson's disease subjects to an age- and sex-matched, balanced cohort of 30 healthy controls.
Blinding	Blinding was not relevant as this study did not include any therapeutic intervention.

Reporting for specific materials, systems and methods

We require information from authors about some types of materials, experimental systems and methods used in many studies. Here, indicate whether each material, system or method listed is relevant to your study. If you are not sure if a list item applies to your research, read the appropriate section before selecting a response.

Materials & experimental systems

n/a	Involved in the study
☒	☐ Antibodies
☒	☐ Eukaryotic cell lines
☒	☐ Palaeontology and archaeology
☒	☐ Animals and other organisms
☒	☐ Clinical data
☒	☐ Dual use research of concern
☒	☐ Plants

Methods

n/a	Involved in the study
☒	☐ ChIP-seq
☒	☐ Flow cytometry
☒	☐ MRI-based neuroimaging

Plants

Seed stocks	N/A
Novel plant genotypes	N/A
Authentication	N/A
